# Research neglect of root system architecture in root and tuber crops important for Sub-Saharan African Food Security

**DOI:** 10.1371/journal.pone.0357467

**Published:** 2026-09-09

**Authors:** Michael O. Adu, David O. Yawson

**Affiliations:** 1 Department of Crop Science, School of Agriculture, College of Agriculture and Natural Sciences, University of Cape Coast, Cape Coast, Ghana; 2 Centre for Resource Management and Environmental Studies, The University of the West Indies, Bridgetown, St. Michael, Barbados; Federal University of Agriculture and Development Studies, Iragbiji (FUADSI), Nigeria, NIGERIA

## Abstract

**Background:**

Root and tuber crops (RTCs) provide 354 kcal/capita/day across sub-Saharan Africa (17.7% of dietary energy from major crop categories), and several countries derive over 40% of crop calories from cassava, yam, sweet potato, taro, and cocoyam. Despite this, research on the root systems that give rise to their harvested storage organs remains limited and concentrated in regions where RTCs contribute little to food security.

**Methods:**

We combined food supply data for 46 sub-Saharan African countries (2018–2022) with a systematic bibliometric analysis of 893 RTC publications (1939–2025; 148 specifically on root system architecture, RSA), a Root Crop Dependency Index (RCDI), and scenario-based economic models, testing whether research attention is inversely related to food-security importance and whether targeted investment could yield measurable returns.

**Results:**

Observed results: Sub-Saharan Africa accounts for only 9.5% of global research output on root system development, despite hosting 60% of cassava production and most yam cultivation; research is concentrated in Asia (44% of publications) and North America (16%), inverting the production geography. Research effort prioritises potato (22% of publications, 13% of regional RTC calories), while yams provide 23% of calories but receive only 4% of research attention, and taro and cocoyam receive under 6%; cassava dominates regional diets (175 kcal capita^-1^ day^-1^) but accounts for only 30% of global publications. The most RTC-dependent countries (the Democratic Republic of the Congo, the Republic of the Congo, and Burundi) contribute minimal research output. Modelled projections: Scenario models suggest yield improvements of 12–25% and economic returns of 5:1–10:1, with the highest for the most neglected crops (taro, cocoyam, yam). These are modelled projections under stated assumptions, not empirical yield trials, and require field validation.

**Conclusions:**

Research capacity for crops that sustain African food security is concentrated in regions where these crops contribute minimally to nutrition. This misalignment may reflect institutional path dependence, donor preferences, and the concentration of infrastructure in high-income countries rather than a systematic assessment of food-security priorities. Given their importance to food security, productivity potential, and current neglect, RTCs appear to be promising, underexplored targets for agricultural research investment; confirming the projected returns will require field trials, breeding pipelines, and adoption studies.

## 1 Introduction

Next to cereals, root and tuber crops (RTCs) are the most important dietary energy source worldwide and are key to global food security. In developing countries, RTCs are the primary source of dietary energy for over 2 billion people. In sub-Saharan Africa (SSA), the cultivation and consumption of RTCs such as cassava (*Manihot esculenta*), yam (*Dioscorea* spp.), sweet potato (*Ipomoea batatas*), taro (*Colocasia esculenta*), and cocoyam (*Xanthosoma sagittifolium*) are critical for food security and livelihoods [1–3]. In most parts of SSA, these crops account for the largest share of cultivated land and of harvested food. The predominance of RTCs in SSA could be attributed to their resilience to climate variability, capacity for year-round production, and adaptability to marginal soils and low-input production systems, making them particularly suited to smallholder farming systems [4,5]. Yet, parts of SSA face acute and chronic food insecurity, compounded by worsening climate variability, recurrent drought, degraded soils, and the limited adaptive capacity of smallholder-dominated farming systems, a set of structural vulnerabilities that are widespread across the developing tropics but most severe in SSA.

This paper concerns a double neglect: root and tuber crops are comparatively neglected within staple-crop research, and within that limited research, root system architecture (RSA), the trait complex most relevant to their productivity, is especially overlooked. Root system architecture (RSA) fundamentally determines plant performance by influencing soil resource acquisition, stress tolerance, and crop productivity [6,7]. The structure and distribution of crop root systems determine how efficiently plants extract water and nutrients from soil. Roots that penetrate deeper access water during drought. Roots that branch extensively explore larger volumes of soil for nutrients. These characteristics can be modified through breeding to improve productivity under the low-input conditions typical of smallholder African agriculture [8,9]. Research on cereals has demonstrated the potential for RSA-based improvements, with targeted breeding for root traits resulting in enhanced drought tolerance, improved nutrient use efficiency, and increased yield stability under stress conditions [7]. However, knowledge of RSA in RTCs remains limited compared to cereals, despite the importance of belowground organs in determining both crop productivity and storage organ quality [10,11].

The physiological characteristics of RTCs present unique opportunities for RSA research. Root and tuber crops differ from cereals in transforming portions of their root systems into storage organs (tubers or enlarged roots). This developmental process links resource acquisition from the soil directly to economic yield in ways that are absent in grain crops. Understanding these linkages could inform strategies for improving productivity under the nutrient-poor and drought-prone conditions common in African farming systems [11]. In cassava and sweet potato, for example, lateral root development directly influences yield determination, while storage root formation involves specific genetic regulation, including MADS-box transcription factors and KNOX1 activity [10, 11]. Understanding how these developmental processes respond to environmental cues could inform strategies for enhancing productivity and stress tolerance in low-input farming systems.

Recent technological advances hold considerable promise for RSA research in RTCs. High-throughput phenotyping methods, three-dimensional imaging techniques, and non-destructive monitoring systems now enable characterisation of root development patterns that were previously impossible to study [12–15]. Ground-penetrating radar can allow non-destructive assessment of storage root development and architectural traits [16]. Artificial intelligence applications in root image analysis further enhance the potential for high-resolution, high-throughput RSA studies, thereby accelerating breeding programmes and genetic research [17]. Despite these technological capabilities, significant knowledge gaps persist in understanding RSA variation, inheritance, and environmental responses in RTCs. Current limitations include inconsistent terminology across studies, limited field-based research under realistic farming conditions, and insufficient integration of RSA traits with crop improvement programmes [8,11].

Despite these technical advances, many RTCs remain classified as Neglected and Underutilised Species (NUS) in Africa, receiving insufficient research attention relative to their contributions to food security [18]. Historical emphasis on cash crops has led to a systematic underestimation of RTCs’ value in the region, resulting in slower research and development progress than in Asia and Latin America [19]. This neglect is particularly problematic given that RTCs are predominantly cultivated by resource-limited smallholder farmers on marginal soils characterised by nutrient deficiencies and water stress conditions, where optimised root systems could provide substantial productivity benefits. The geographic concentration of RSA research in developed regions, despite the predominance of RTC cultivation in developing countries, further deepens the knowledge deficits. In SSA, research capacity constraints, including limited infrastructure, technical expertise, and funding, restrict the ability of regional institutions to advance root research in RTCs, creating a disconnect between research capacity and crop importance [20,21]. The importance of neglected crops for food security and nutrition is recognised in international agricultural development research [22]. Even so, laudable initiatives such as the African Orphan Crops Consortium (https://africanorphancrops.org/) that focus on neglected species still do not give full attention to root system research. At the same time, research outputs on RSA in RTCs remain fragmented, making it difficult to prioritise research actions to scale impacts.

Agricultural research investment constitutes a policy choice with distributive consequences. Resources allocated to one crop reduce funding available for others, creating winners and losers among farmers and consumers. Although effects are nuanced and context-dependent, in market economies, private-sector research substantially follows commercial incentives [23], concentrating effort on crops with profitable seed markets and intellectual property protection. Public agricultural research ostensibly corrects these market failures by targeting crops and production systems serving resource-poor farmers and consumers [24]. Yet public research institutions face their own distortions. Donor priorities, infrastructure path dependence, and prestige hierarchies within agricultural science create systematic biases that favour certain crops regardless of their importance to the recipient country's food security. Whether these biases produce measurable misalignment between research effort and nutritional priorities remains an empirical question warranting systematic investigation.

This paper tests whether research attention to root and tuber crops aligns with their importance for food security in sub-Saharan Africa. We examine two hypotheses: (H1) that research effort is inversely related to the importance of food security, with neglected crops receiving minimal attention despite high consumption; and (H2) that strategic research investment in these neglected crops could yield measurable returns for food security. Specifically, we: (1) quantify dietary dependence on root and tuber crops across sub-Saharan African regions to establish food security importance; (2) assess current research attention through bibliometric analysis of global publication patterns; (3) test for misalignment between food security importance and research effort; (4) model potential productivity improvements and returns under alternative research investment scenarios; and (5) identify policy interventions addressing institutional barriers to research prioritization in food security crops.

This study asks a single question: does research attention to root system architecture (RSA) in root and tuber crops match the food-security importance of those crops in sub-Saharan Africa? The gap is therefore one of crop and trait prioritisation; Africa's research capacity constraints are a contributing cause, not the gap itself. Each analysis that follows addresses one part of this question. We employ food-supply and dependency data to establish importance, a bibliometric analysis to measure research attention, and a scenario-based model to illustrate what realigning attention could yield. In this study, RSA refers to the spatial configuration of the root system and is treated as a defined subset of the broader root-system research literature, rather than as a synonym for root-system development.

## 2 Materials and methods

### 2.1 Regional analysis of dietary dependence on root and tuber crops

Five-year food supply data for 46 sub-Saharan African countries were obtained from the Food and Agriculture Organisation (FAO) FAOSTAT database, covering the period 2018–2022. Countries were categorised into four regions: Central Africa (n = 8), East Africa (n = 17), Southern Africa (n = 5), and West Africa (n = 16). Food supply statistics were expressed as kilocalories per capita per day (kcal/capita/day). Agricultural commodities were classified into three categories based on botanical and nutritional characteristics: (1) Root and tuber crops including cassava and products, potatoes and products, sweet potatoes, yams, and other roots; (2) Cereals comprising wheat and products, rice and products, barley and products, maize and products, rye and products, oats, millet and products, sorghum and products, and other cereals; and (3) Legumes including beans, peas, pulses and products, soybeans, and groundnuts.

Data quality control procedures included removing records with missing or negative values, standardising country names, validating temporal consistency, and verifying unit consistency. Countries with incomplete data series (more than 20% missing values) were excluded from temporal analyses but retained for cross-sectoral comparisons. Regional aggregation used unweighted country means as the primary basis, giving each country equal weight regardless of population size; population-weighted means were also computed as a robustness check and are reported in the Supporting Information (S3 Table). A Root Crop Dependency Index (RCDI) was developed to quantify reliance on root and tuber crops, as defined in Eq 1. The RCDI expresses the combined dietary energy from all root and tuber crops as a share of the total dietary energy supply. We preferred it to simpler measures because it aggregates the full RTC portfolio rather than a single dominant staple, and because expressing dependence as a share of total dietary energy, rather than as absolute consumption or production tonnage, makes it comparable across countries with different total intakes and captures relative reliance, the quantity of policy interest. It is a transparent, fully reproducible descriptive index rather than a latent construct requiring psychometric validation; we therefore do not claim formal validation, but report its robustness to the classification threshold (S1 Table) and note that its rankings recover countries independently known to depend heavily on RTCs (e.g., the Democratic Republic of the Congo). Regional RCDI values were computed on the same unweighted basis.


$$\begin{array}{c} RCDI=\left(\frac{\text{Root }\&\text{ Tuber Calories }}{\text{Total Calories from Crops}}\right)\times \text{1}\text{0}\text{0} \end{array}$$ (1)

Countries were classified into dependency categories: High (≥40%), Moderate (20–39%), Low (10–19%), and Minimal (<10%). Crop portfolio diversity was assessed using the Shannon-Weaver diversity index (Eq 2).


$$\begin{array}{c} {\text{H}}^{\prime }=-\sum \left(pi\times \text{ln}(pi)\right) \end{array}$$ (2)

where *pi* represents the proportional caloric contribution of each crop category. Temporal stability was evaluated using the coefficient of variation and trend analysis, as well as Pearson correlation coefficients between year and consumption values for each country-crop combination.

### 2.2 Bibliometric analysis of root system architecture research

A bibliometric analysis was conducted to assess the current state of RSA research in RTCs worldwide, with a specific focus on contributions from SSA. The analysis employed systematic literature search protocols, standardised inclusion criteria, and quantitative assessment techniques to evaluate research patterns, geographic distribution, and thematic focus across the scientific literature.

### 2.3 Literature search strategy

Systematic literature searches were conducted using two complementary databases: Scopus and Google Scholar. Scopus and Google Scholar were chosen as complementary sources. Scopus provides broad, well-indexed coverage of agricultural and plant-science journals with reliable, exportable metadata, while Google Scholar captures grey literature, theses, conference proceedings, and regionally published work that indexed databases under-represent, a particular concern for sub-Saharan African root and tuber crop research, much of which appears in journals not indexed by Scopus or Web of Science.

The search query was designed to capture publications addressing root system research in major root and tuber crops: {Root system architecture} OR {root morphology} OR {root trait*} OR {root phenotyping} OR {root development} OR {root system*} OR {plant root*} AND cassava OR yam* OR sweetpotato* OR {sweet potato*} OR potato* OR cocoyam* OR taro OR {root crop*} OR {tuber crop*} OR {root and tuber crop*}. For Google Scholar, which returns results in relevance-ranked order and does not support exhaustive structured export, records were screened sequentially in that order rather than truncated at a fixed number. Screening continued until successive results no longer returned publications relevant to root system research in root and tuber crops; this point of diminishing relevance was reached at 529 records, all of which were exported and screened. We acknowledge that this relevance-based stopping rule is necessarily somewhat subjective.

Scopus was searched on 17 April 2025 (a core string and a refined variant) and again on 21 April 2025 with a string extended to include potato terms; Google Scholar was searched on 21 April 2025. Records from all searches were combined into a single file, retaining the source database and search date for each record, and duplicates, within Scopus and between Scopus and Google Scholar, were removed by matching on title and, where available, DOI. Title and abstract screening and the subsequent categorisation were performed independently by two researchers, with the lead author adjudicating disagreements and making the final classification; formal inter-rater agreement (Cohen’s κ) was not computed. Records reporting more than one crop were assigned to their primary focus crop, and records that could not be reliably classified were excluded at a final verification step. The flow of records from identification to inclusion is summarised in a PRISMA 2020 flow diagram (S1 Fig). The bibliometric dataset, the country-level Root Crop Dependency Index, and the FAOSTAT food-supply and production data underlying all analyses are provided in S1 Dataset.

### 2.4 Inclusion and exclusion criteria

The analysis employed a hierarchical screening approach, with distinct inclusion criteria for each analytical phase. Initial screening for global research assessment included publications meeting the following criteria: (1) peer-reviewed journal articles, conference proceedings, or book chapters published in any year up to May 2025; (2) primary focus on one or more major RTCs (cassava, sweet potato, potato, yam, taro, cocoyam); (3) content addressing any research aspect related to these crops, encompassing agronomic, physiological, genetic, pest management, post-harvest, and root system studies; (4) publications in English language; and (5) accessibility of complete bibliographic metadata including title, authors, publication year, and institutional affiliations.

This dataset enabled the assessment of Africa’s contribution to global RTC research and the distribution of research across all thematic domains. Subsequently, a refined subset was extracted specifically for RSA analysis, applying additional filtering criteria: (6) explicit focus on root system components, architecture, morphology, development, or related belowground traits as determined through title and abstract screening; and (7) inclusion of root system-specific terminology or methodologies. Exclusion criteria comprised: (1) review articles and meta-analyses to focus on primary research contributions; (2) publications focusing exclusively on non-target crops (cereals, legumes, or horticultural crops without root/tuber storage organs); (3) studies addressing only above-ground plant components without root system relevance; (4) duplicate entries across databases; (5) gray literature including technical reports, theses, and non-peer-reviewed publications; and (6) publications lacking sufficient metadata for geographic or thematic classification.

### 2.5 Data extraction and standardisation

Data extraction protocols included bibliographic information (title, publication year, and journal), geographic indicators (where the research was conducted, but if not explicitly stated, country of the first author’s institutional affiliation), primary crop species focus, and research thematic area, as determined through content analysis of the title and abstract. Research areas were classified into 13 standardised categories: (1) Root System Architecture, encompassing studies specifically addressing root morphology, architecture, traits, phenotyping, and development; (2) Genetics/Molecular Biology, including molecular markers, quantitative trait loci, genomics, and genetic improvement studies; (3) Biochemistry/Physiology, covering metabolic processes, enzyme activities, and physiological responses; (4) Nutrition/Fertilisation, including nutrient uptake, fertiliser response, and mineral nutrition studies; (5) Pest and Disease Management, encompassing pathogen resistance, pest control, and plant protection research; (6) Stress Response, covering drought, salinity, temperature, and other abiotic stress research; (7) Breeding/Genetics, including conventional breeding approaches and variety development; (8) Post-harvest/Processing, addressing storage, processing technologies, and value addition; (9) Agronomy/Management, covering cultivation practices, field management, and cropping systems; (10) Soil/Environment, encompassing rhizosphere interactions, microbial associations, and soil-plant relationships; (11) Biotechnology, including tissue culture, genetic engineering, and biotechnological applications; (12) Quality/Composition, addressing nutritional content, chemical composition, and food quality parameters; and (13) Other, encompassing studies that did not fit clearly into the above categories.

Country affiliations were standardised to address variations in institutional naming conventions and encoding inconsistencies through text-processing procedures. Standardisation protocols included removing special characters, consolidating variants of country names (e.g., “USA,” “United States,” “United States of America”), and manually verifying ambiguous cases. Countries were subsequently aggregated into geographic regions based on United Nations geoscheme classifications: Sub-Saharan Africa, North Africa and the Middle East, East Asia, South and Southeast Asia, Europe, North America, Latin America, and Oceania. The classification of the crops employed standardised botanical nomenclature, aggregating species variants and product derivatives. Major categories included cassava (*Manihot esculenta* and products), sweet potato (*Ipomoea batatas*), potato (*Solanum tuberosum* and products), yam (*Dioscorea* spp.), taro (*Colocasia esculenta*), and cocoyam (*Xanthosoma sagittifolium*). Publications addressing multiple crops were classified by the primary focus species, as determined through systematic title and content analysis.

### 2.6 RSA-focused bibliographic analyses

The bibliographic search identified 148 studies focusing on RSA in root and tuber crops from 1985 to 2025. Studies were classified by crop type (cassava, sweet potato, potato, yam, taro), geographic origin, research context (greenhouse, field, laboratory), and data-acquisition method. Methods for extracting root traits were standardised into categories: manual counting, digital imaging, scanner-based analysis, microscopy (light and electron), manual and digital hybrid approaches, anatomical analysis, and grid intersection methods. RSA data were categorised into seven trait classes: morphological (length, diameter, volume), topological (branching patterns, root numbers), storage root traits (tuber-specific parameters), anatomical (tissue structure), dynamic (growth rates), geometrical (spatial distribution), and root hair characteristics.

### 2.7 Analytical framework

Temporal analysis divided the study period into discrete time intervals (pre-1980, 1980–1989, 1990–1999, 2000–2009, 2010–2019, 2020-present) to assess patterns of research evolution and identify periods of accelerated research activity. The focus on root system architecture was determined through a keyword analysis of publication titles, with binary classification (RSA-focused vs. non-RSA-focused) applied to each record based on the presence of specific terminology related to root system structure, development, or architectural traits.

We operationalised a “double neglect” concept through quantitative assessment of two independent dimensions: (1) Neglected and Underutilised Species (NUS) status, based on established classifications in literature, with yam, taro, and cocoyam designated as NUS crops; and (2) Root system architecture research intensity, calculated as the proportion of publications within each crop category specifically addressing RSA themes relative to total research volume for that crop.

### 2.8 Root system architecture improvement modelling and impact assessment

#### 2.8.1 Theoretical framework development.

We developed a theoretical framework to estimate the potential yield improvements achievable through RSA optimisation across six RTCs important to food security in SSA. The framework combines established plant physiological relationships with breeding theory to project impacts under different research investment scenarios. We acknowledge this represents a modelling exercise rather than empirical research, with assumptions detailed below.

#### 2.8.2 Crop selection baseline data, root system architecture trait framework, and assumptions.

We selected six crops based on regional production importance: cassava (*Manihot esculenta*), sweet potato (*Ipomoea batatas*), yam (*Dioscorea* spp.), potato (*Solanum tuberosum*), taro (*Colocasia esculenta*), and cocoyam (*Xanthosoma sagittifolium*). Production data were obtained from FAOSTAT (averages for 2018–2022). Caloric availability (C) was calculated with Eq 3.


$$\begin{array}{c} C=\frac{\text{P }\times \text{ D }\times \text{ 1000}}{\text{Pop }\times \text{ 365}}\text{,} \end{array}$$ (3)

where C represents the estimated caloric availability per capita per day from each crop across SSA, calculated from production data under the simplifying assumption of negligible net exports. It serves as a proxy for the crop’s contribution to the aggregate dietary energy supply across SSA. Regional consumption figures reported in the Results are derived directly from FAOSTAT food supply statistics rather than from Eq 3, which is used only for the crop-level investment modelling; P = production (Mt), D = caloric density (kcal kg^-1^), for each crop obtained from FAO food composition databases, and represent energy content per kilogram of fresh weight. Values were adjusted to account for typical post-harvest losses in SSA smallholder systems, which range from 20–30% depending on crop type and storage conditions, with Pop denoting the sub-Saharan African population (1.2 × 10⁹).

Current attention to RSA research was estimated using the proportion of crop research publications that focus on root system traits. We acknowledge that this provides only an approximate measure of actual research investment, but it represents the best available proxy for comparative research intensity across crops. We selected six root characteristics for modelling based on their documented effects on crop performance under stress conditions common in African agriculture. Deeper rooting enables access to water during dry periods. Higher density of fine absorptive roots improves nutrient capture in depleted soils. Air spaces in root tissue (aerenchyma) prevent roots from suffocating in waterlogged fields. The ratio of root mass to shoot mass indicates resource allocation between aboveground and belowground structures. These traits can be modified through conventional breeding, as demonstrated in cereal crop improvement programmes.

#### 2.8.3 Ideotype development.

We developed theoretical ideotypes for four stress conditions by applying multipliers to baseline trait values. Thus, the ideotype value for each stress condition was calculated using a multiplicative improvement model that integrates baseline crop performance with stress-specific enhancement factors. This approach assumes that responses to genetic improvement are independent and that physiological benefits scale linearly, consistent with quantitative genetic models of complex traits. Specifically, the assumptions here were that: (i) trait improvements follow linear responses, (ii) multiple traits can be optimised simultaneously without major trade-offs, and (iii) optimal combinations remain stable across environments. These assumptions likely overestimate the achievable improvements but provide useful upper bounds on the theoretical potential. Ideotype multipliers were constrained to documented ranges of genotypic trait variation in the peer-reviewed literature. However, we acknowledge that direct evidence of breeding for achievable improvements in RTCs under field conditions remains limited. For drought tolerance, we modeled varieties with 50% deeper rooting and 40% greater allocation of plant biomass to roots relative to current cultivars. These improvement levels reflect documented variation among existing wheat and maize varieties, suggesting biological feasibility. To improve nutrient efficiency in depleted soils, we modelled a 45% increase in root surface area via finer, more extensively branched root systems. For waterlogging tolerance, we modelled an 80% expansion of air spaces in root tissue to maintain oxygen supply when soils are flooded. These improvement targets represent conservative estimates based on natural variation documented in related crop species.

The 1.5 × rooting-depth multiplier was set to reflect a moderate enhancement, but available evidence indicates that cassava is naturally shallow-rooted, with roots rarely extending below 105 cm and relatively small genotypic variation in rooting depth [25,26], suggesting that our assumed enhancement may overestimate achievable gains. Drought adaptation in cassava relies more on physiological water extraction efficiency and horizontal root spread than on increased rooting depth [27] The 1.4 × root: shoot ratio enhancement reflects moderate biomass reallocation toward roots, though wheat studies show variable responses to drought stress, with some genotypes increasing root: shoot ratios by up to 75% while others show reductions of 14%, and absolute ratios ranging from 0.30 to 1.48 across genotypes [28,29]. Root hair density enhancement (1.45×) is supported by documented 2-fold genotypic variation in root hair length and density in rice, wheat, and soybean under low-phosphorus conditions, with superior genotypes increasing total root surface area by 31% through root hairs and contributing substantially to phosphorus uptake efficiency [30–32]. Lateral branching enhancement (1.35×) reflects demonstrated benefits of increased lateral root density for phosphorus acquisition in maize, where greater branching density significantly improves phosphorus uptake, biomass accumulation, and yield in low-phosphorus soils [33,34]. Aerenchyma formation enhancement (1.8×) is supported by evidence that waterlogging-tolerant genotypes develop larger, more rapidly formed aerenchyma than sensitive genotypes across wheat, barley, maize, and cotton, with barley genotypes carrying aerenchyma-formation quantitative trait loci showing 1.8 t/ha yield increases under field waterlogging compared to non-carriers [35–38]. Disease resistance multipliers (1.1–1.3×) follow the multiplicative survival model approach demonstrated by Grimmer et al. [39], in which resistance effectiveness from multiple loci is expressed as proportional severity ratios, and predicted severity is calculated as the product of these proportions. These were set conservatively, given the limited direct evidence of heritability for the disease-resistance trait in RTCs under field conditions. We emphasise that these multipliers represent working assumptions based primarily on cereal and legume evidence, with direct validation for RTCs under smallholder farming conditions in sub-Saharan Africa representing a critical research priority that this analysis aims to highlight. The optimisation model was defined as:


$$\begin{array}{c} I_{trait,stress}=B_{trait}\times M_{trait,stress} \end{array}$$ (4)

where $I_{trait,stress}$ is the ideotype value for a specific trait under defined stress conditions, $B_{trait}$represents baseline performance normalised to 100%, and $M_{trait,stress}$represents stress-specific multipliers derived from literature and documented breeding programme outcomes.

#### 2.8.4 Yield improvement modelling.

Expected yield improvements were calculated using a probability-weighted approach that accounts for both biological potential and the likelihood of research development. We modelled five improvement mechanisms: enhanced nutrient uptake (15–35% yield gain), improved drought tolerance (20–60% gain), waterlogging tolerance (15–65% gain), disease resistance (15–45% gain), and lodging resistance (8–28% gain). The assumptions here were that: (i) benefits are additive across mechanisms, (ii) improvements translate directly to yield under farmer conditions, and (iii) current genotype-by-environment interactions don’t limit trait expression. Improvement ranges were based on documented breeding successes in cereals and limited data from root crops; however, we acknowledge substantial uncertainty in extrapolating these findings to diverse African farming systems. The wide ranges reflect this uncertainty rather than precise estimates. Thus, this methodology recognises that theoretical improvements must be tempered by realistic assessments of achievability within existing research and development frameworks. The prediction model was formulated as:


$$\begin{array}{c} \text{Expected Improvement}=\sum \left(\text{Mechanism Improvement }\times \text{ Achievement Probability}\right) \end{array}$$ (5)

where Expected Improvement represents the probability-adjusted yield gain, Mechanism Improvement quantifies the biological potential for enhancement through specific physiological pathways, and Achievement Probability reflects the likelihood of successful implementation based on research capacity and investment levels.

### 2.9 Achievement probability assessment, economic modelling, and scenario development

We estimated breeding success probabilities for each crop using the relationship between prior research investment and documented breeding outcomes reported in the literature. Crops with established research infrastructure, including characterised germplasm collections, genetic mapping populations, and available RSA phenotypic datasets (cassava, sweet potato, potato), were assigned probabilities of 70–85%, reflecting the documented association between research capacity and variety development success in international breeding programmes [40,41]. For moderately researched crops such as yams, where germplasm characterisation exists but RSA-specific datasets remain limited, we assigned probabilities of 50–65%. For under-researched crops (taro, cocoyam), for which RSA data are absent and breeding infrastructure is minimal, we assigned 30–50%. These estimates are consistent with the range of breeding success rates reported across different crop improvements in Alston et al. [42], who documented that investment in data-poor research environments is associated with substantially higher variance and lower expected returns than investment in crops with established research foundations. We acknowledge that these remain approximations rather than empirically derived values, and that factors such as institutional capacity, political economy, and seed systems can introduce additional uncertainty. Scenario rankings are robust to modest variations in these probabilities, as the key comparative message holds across a plausible range. We developed five investment scenarios ($0-400M USD over 6–15 years) representing different research commitment levels. Food security impacts were calculated using a simplified model:


$$\begin{array}{c} \text{Additional Calories }=\frac{\text{Yield Improvement }\times \text{ Coverage }\times \text{ Current Production }\times \text{ Caloric Density}}{\text{Population }\times \text{ 365}} \end{array}$$ (6)

Coverage denotes the proportion of farmers adopting improved varieties; Current Production reflects baseline crop production; Caloric Density indicates crop-specific calories per kg; and population denotes the sub-Saharan African population (1.2 × 10⁹). We assumed static population estimates and did not incorporate demographic projections, shifting consumption patterns, or detailed post-harvest loss adjustments. The model assumes a direct translation of increases in production into caloric availability, which likely overestimates the actual impacts on food security. Some economic assumptions were also made, but we acknowledge that these assumptions may not hold under changing market conditions or climate scenarios. The economic assumptions were that (i) Farmgate prices remain stable at ~$200/Mt, (ii) adoption follows logistic curves typical of variety adoption, (iii) no market saturation effects, and (iv) benefits persist over variety lifespans. Return-on-investment calculations used standard cost-benefit analysis but excluded many difficult-to-quantify benefits (environmental services, nutritional quality improvements, climate-adaptation value), likely underestimating total returns.

### 2.10 Statistical analysis

Descriptive statistics were calculated for continuous variables, and frequency distributions were constructed for categorical variables. Temporal trends in RSA research were analysed using LOESS smoothing, and cross-tabulation was used to examine relationships among trait categories, crop types, and methodological approaches. For theoretical modelling, expected yield improvements were calculated by summing the individual mechanism improvements, each weighted by its probability of achievement. Regional comparisons used ranking and percentage calculations, whereas economic projections employed deterministic models with point estimates of return-on-investment ratios and food security impacts. Research prioritisation analysis utilised a two-dimensional matrix plotting current research investment against improvement potential scores. All analyses were performed in R (version 4.3.0) [43] using tidyverse, dplyr, and ggplot2 packages.

## 3 Results

### 3.1 Regional variation in root and tuber crop consumption

Root and tuber crops (excluding cocoyam) supply 354.3 kcal capita^-1^ day^-1^ to SSA consumers, representing 17.7% of the recommended daily caloric intake (Table 1). Cassava dominates the regional dietary energy supply, providing 175.5 kcal capita^-1^ day^-1^ from 15.8 million metric tons of annual production, followed by yam (83.0 kcal capita^-1^day^-1^ from 7.5 million metric tons). Total production across five documented crops amounts to 32.0 million metric tons annually, from 3.6 million hectares. Cocoyam is excluded here because it is not reported separately in the FAOSTAT food-supply data (it falls within “Roots, Other”) and is absent from the production data; its prioritisation therefore rests on its documented regional role rather than on complete statistics.

**Table 1:** Production and caloric availability of root and tuber crops in sub-Saharan Africa (FAOSTAT averages, 2018–2022). “RSA research proportion (%)” is the share of the 148 RSA-focused publications in our bibliometric dataset that concern each crop; it is not a measure of financial investment.

Crop	Production(Mt yr^-1^)	Yield(t ha^-1^)	Area(Mha)	Calories(kcal capita^-1^day^-1^)	RSA Research Proportion (%)
Cassava	15.8	8.3	1.916	175.5	29.7
Yam	7.5	8.5	0.881	82.8	4.7
Potato	4.2	18.5	0.226	46.5	23.0
Sweet Potato	2.4	7.3	0.324	26.2	39.9
Taro	2.1	7.4	0.282	23.3	0.7
Cocoyam	NA	NA	NA	3.4	0

**Fig 1:**
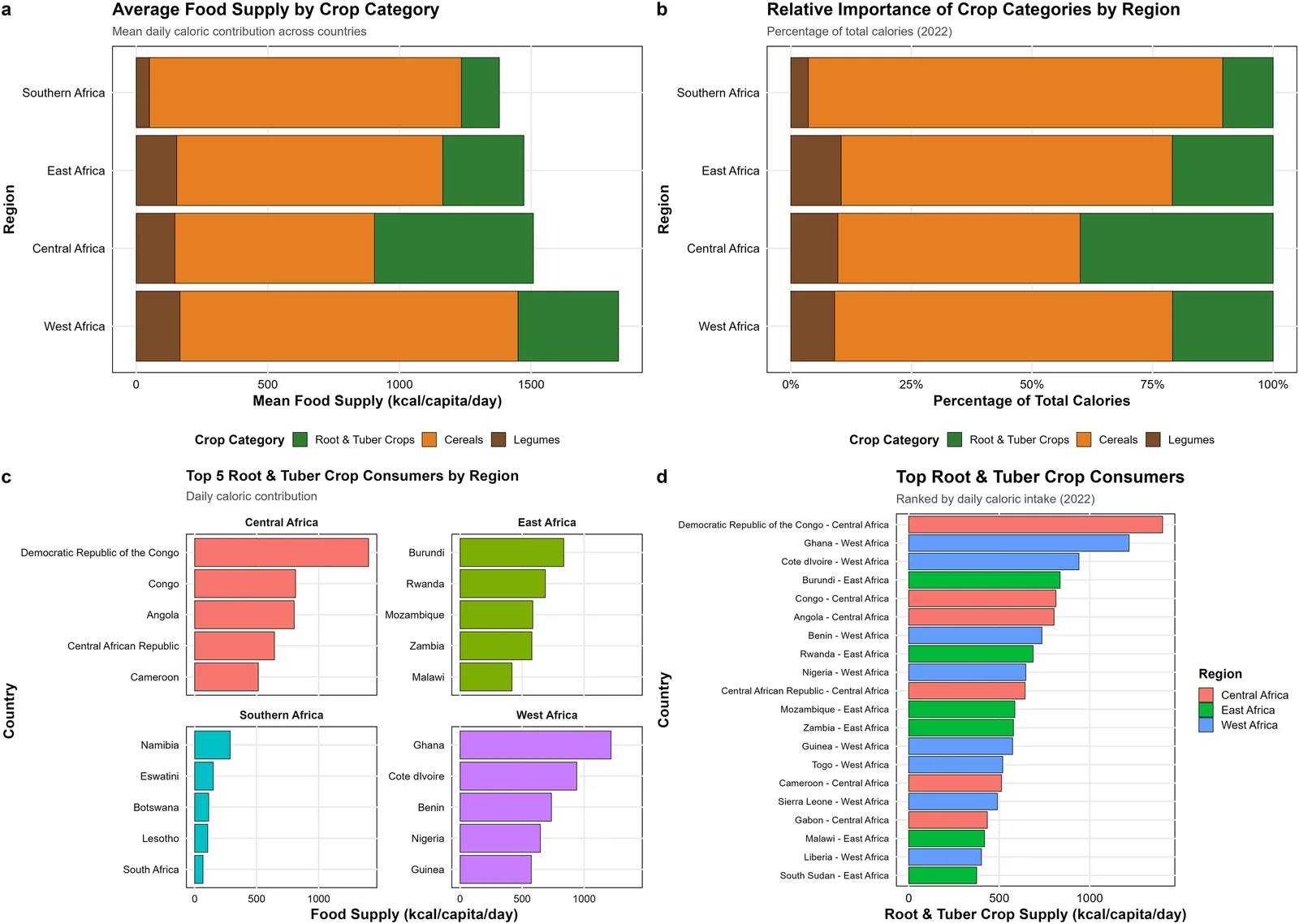
Regional food supply across sub-Saharan Africa (46 countries, 2018–2022 averages). (A) Mean daily caloric contribution by crop category per region (kcal/capita/day). (B) Calories from major crop categories by region, as a percentage of the total from cereals, RTCs and legumes. (C) Top five RTC consumers per region. (D) Top 20 countries by RTC consumption (kcal/capita/day), colour-coded by region.

Interregional differences in RTC dependence were identified (Fig 1A). Central Africa had the highest mean consumption at 603.5 kcal/capita/day, which is 4.2 times greater than that of Southern Africa (144.2 kcal/capita/day). West Africa (381.8 kcal/capita/day) and East Africa (306.9 kcal/capita/day) recorded intermediate consumption levels, with West Africa consuming 2.7 times as much as Southern Africa. Proportional analysis of major crop categories showed that RTCs accounted for 40.0% of total calories from major crops in Central Africa, compared with 10.4% in Southern Africa, a 3.8-fold difference (Fig 1B). East Africa and West Africa showed equivalent proportional dependence at 20.8%. Cereals dominated the food supply across all regions, ranging from 50.2% of major crop calories in Central Africa to 85.9% in Southern Africa.

These regional means weight each country equally. We also computed population-weighted means, using national populations as weights (S3 Table). Weighting raises the estimates rather than lowering them: the sub-Saharan African mean rises to approximately 491 kcal capita^-1^ day^-1^, and the Central and West African means to roughly 994 and 571 kcal capita^-1^ day^-1^ respectively, because the most populous countries, notably Nigeria and the Democratic Republic of the Congo, are also among the most root-and-tuber-dependent. The unweighted figures reported above are therefore the more conservative estimate of dietary importance.

### 3.2 Country-level root and tuber crop consumption patterns

The Democratic Republic of the Congo had the highest consumption at 1,437.6 kcal/capita/day (Fig 1C and 1D). Among the top five regions by consumption, Central Africa recorded the widest range (507-1,438 kcal/capita/day), while Southern Africa had the narrowest (85–205 kcal/capita/day). The consumption differential between the highest (Democratic Republic of the Congo) and lowest (South Africa, 61 kcal/capita/day) consumers was 23.6-fold. Central Africa accounted for 5 of the top 20 global consumers, despite representing only 17.4% (8/46) of the total country sample.

### 3.3 Root crop dependency index classification

**Fig 2:**
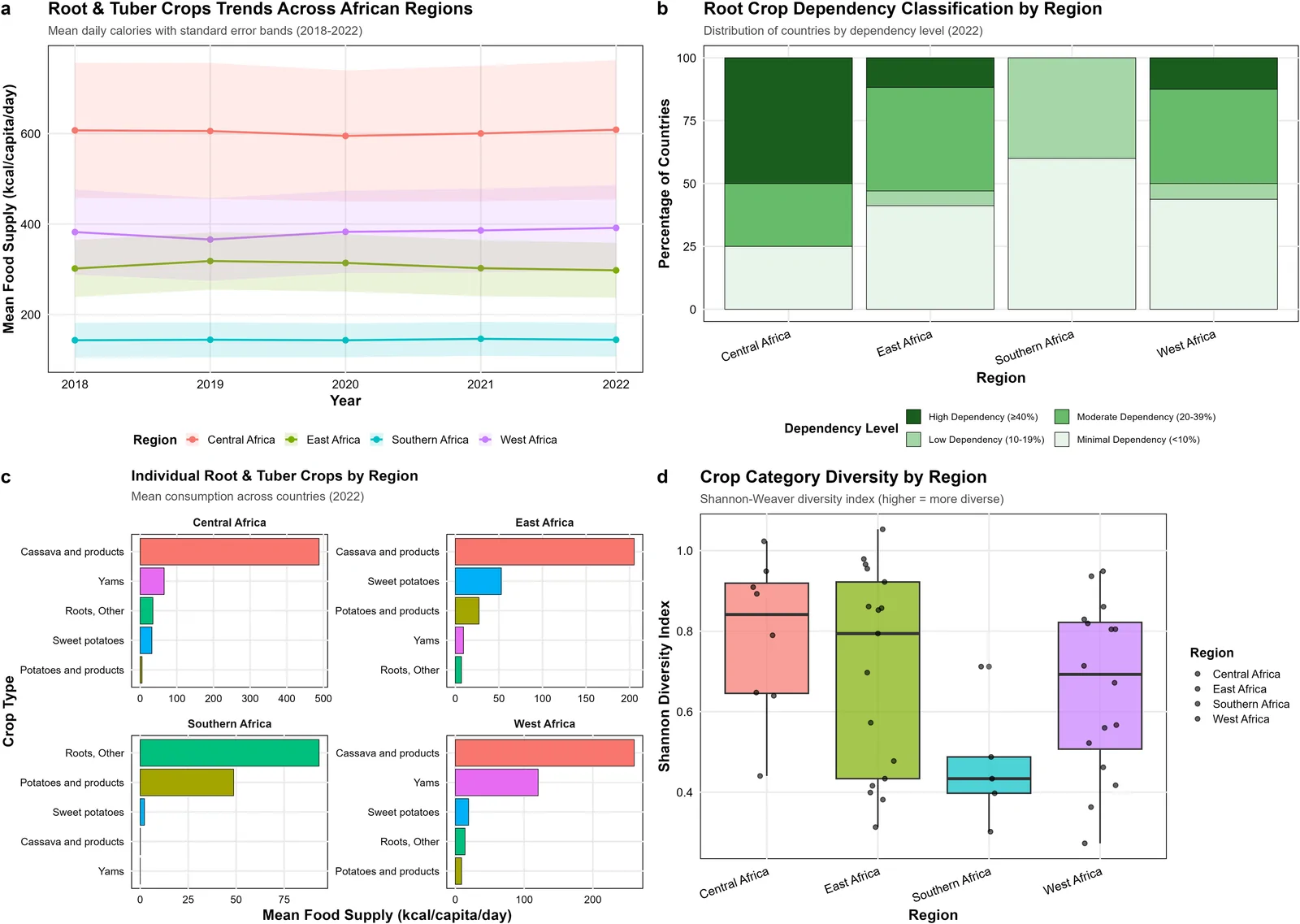
Root crop dependency and temporal trends (2018–2022). (A) Mean RTC consumption by region over time, with standard-error bands. (B) Distribution of countries across Root Crop Dependency Index categories, by region. (C) Species-specific RTC consumption by region. (D) Shannon–Weaver diversity index for the crop-category portfolio, by region.

As noted in the Methods, the RCDI is a descriptive index rather than a validated instrument; its robustness to the classification threshold is shown in S1 Table. The RCDI classified countries into four categories of dependency (Fig 2A). Eight countries (17.4%) exhibited high dependence (≥40%), with Central Africa accounting for 50% of this category, even though it accounts for only 17.4% of the total number of countries. Fifteen countries (32.6%) demonstrated moderate dependence (20–39%), while 19 countries (41.3%) showed minimal dependence (<10%) on RTCs. Regional RCDI statistics established that Central Africa’s mean of 39.0% exceeded the overall mean (22.3%) by a factor of 1.7. The highest individual country RCDI reached 78.0% (Democratic Republic of the Congo), while the lowest recorded was 1.1%. Central Africa recorded the greatest RCDI variability (SD = 25.0), spanning from 7.7% to 78.0%.

### 3.4 Temporal stability and crop diversity

We observed stable RTC consumption patterns across all regions for the study period of 2018–2022 (Fig 2A). West Africa recorded the largest absolute change (+9.2 kcal/capita/day), representing a 2.4% increase. The other regions changed only marginally. Cassava dominated RTC consumption in Central, East and West Africa (Fig 2C). Southern Africa demonstrated a different pattern, with “Roots, Other” (i.e., other root crops) contributing 90 kcal/capita/day and potatoes 55 kcal/capita/day. Yams contributed substantially in West Africa (146 kcal/capita/day) but minimally elsewhere. Shannon-Weaver diversity analysis identified Central Africa as the most diverse region (H’ = 0.780), exceeding Southern Africa (H’ = 0.471) by a factor of 1.7 (Fig 2D). East Africa and West Africa showed intermediate diversity.

### 3.5 Research attention patterns

**Fig 3:**
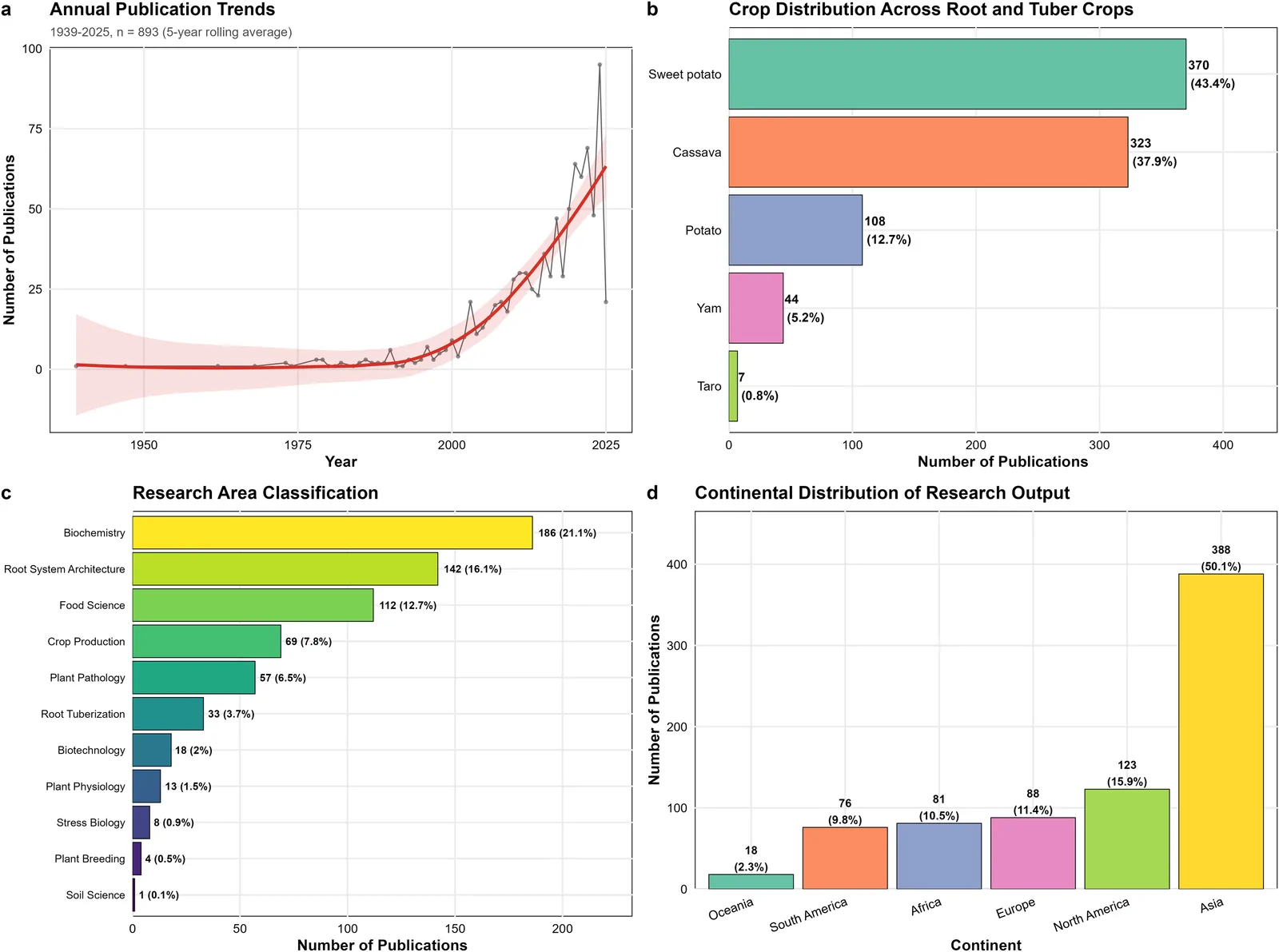
Overview of root and tuber crop research. (A) Annual publications, 1939–2025, with 5-year rolling average (red line). (B) Distribution by crop. (C) Geographic distribution. (D) Distribution by continent.

**Fig 4:**
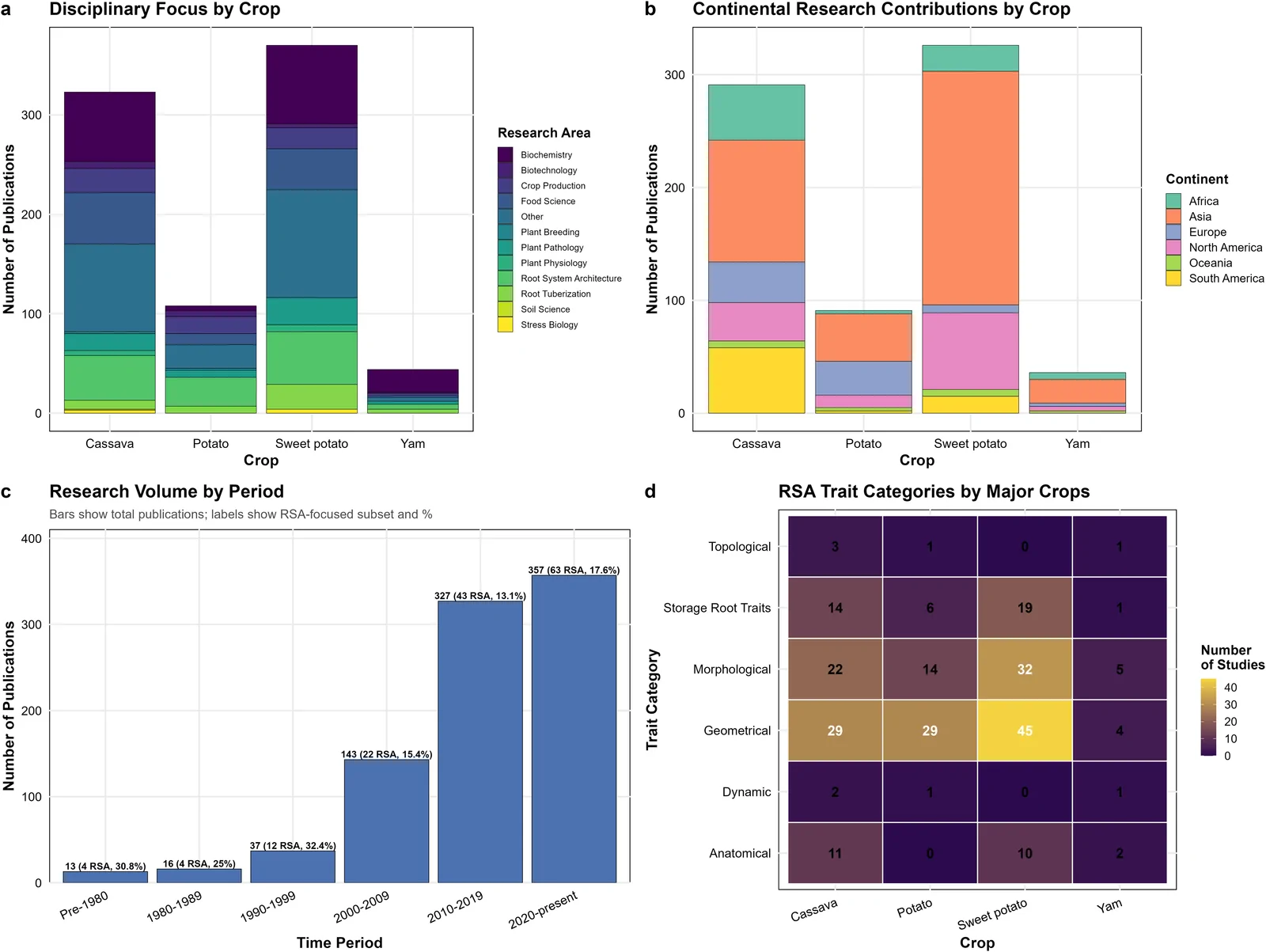
Research patterns and temporal dynamics in RTC studies. (A) Disciplinary focus by crop. (B) Continental research contributions by crop. (C) Publication counts by period, with the percentage indicating RSA research. (D) RSA trait categories (anatomical, dynamic, geometrical, morphological, storage-root, topological) measured per crop, for cassava, potato, sweet potato and yam.

An overview of root and tuber crop studies is presented in Fig 3. Our bibliometric analysis identified 893 publications on root and tuber crops between 1939 and 2025, with research output accelerating after 2000. Sweet potato and cassava together account for 77.6% of research attention (370 and 323 papers, respectively), while yams (44 papers) and taros (7 papers) remain understudied. Of the 893 publications, 148 (17%) specifically addressed root system development. Asia accounted for 48% of root research. Sub-Saharan Africa contributed only 9.5% of the global root-system research output. Research within the root system subset focused primarily on potato (32% of crop-specific studies) and sweet potato (16%), with cassava at 14% and yam at 16%. Patterns of research and temporal dynamics in root and tuber crop studies are presented in Fig 4. The temporal pattern showed research effort expanding across all crops over recent decades, but proportional attention to root systems declined from 31% pre-1980 to 18% currently. Most studies examined basic root structure through manual measurement or imaging (60% of all root research), while traits specific to storage organ development received minimal attention (10%).

### 3.6 RSA research intensity, double neglect, and geographic distribution

**Fig 5:**
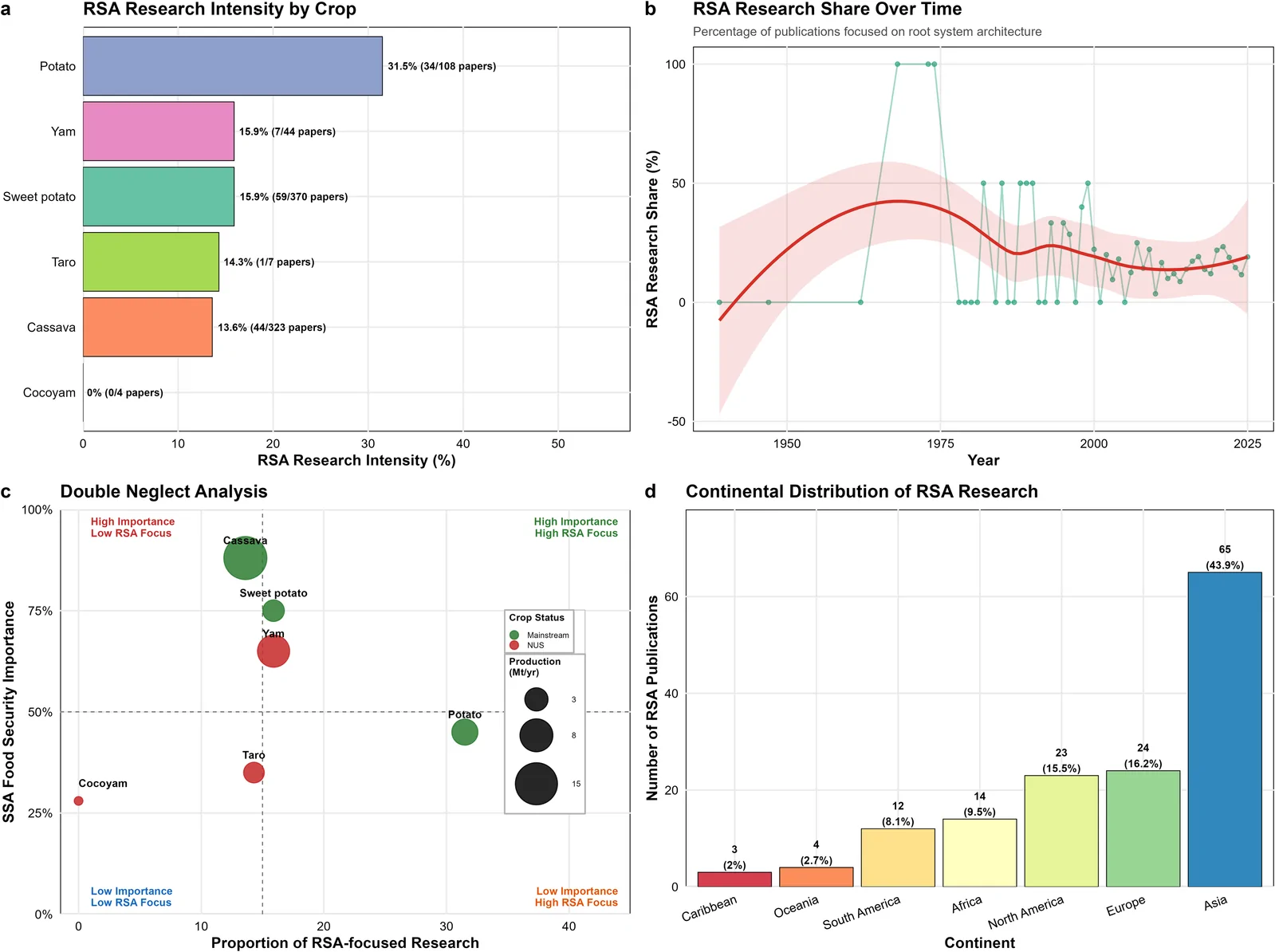
RSA research intensity and the double-neglect pattern. (A) RSA research intensity by crop (percentages and counts). (B) RSA research share over time, with LOESS smoothing. (C) RSA research proportion versus sub-Saharan food-security importance, by crop. (D) RSA research by continent (counts and percentages).

Based on the number of eligible papers per crop, RSA research intensity varied substantially, with potato achieving the highest proportion at 32% (34/108 papers, Fig 5A). This distribution represents a 2.2-fold (32% versus 14%) difference between the highest and lowest RSA research intensities. Temporal analysis showed variable proportions of RSA research, with no consistent directional trend (Fig 5B). There was a peak in RSA activity during the 1980s, followed by a gradual decline and recent stabilisation around 20–25%.

The double-neglect analysis positioned crops according to the RSA research proportion and their food security importance in SSA (Fig 5C). Cassava, sweet potato, and yam clustered in the high-importance, low-RSA-focus quadrant. Potato occupied the low-importance, high-RSA-focus position, while taro and cocoyam fell into the low-importance, low-RSA-focus category. Research volume indicators showed that sweet potato commanded the largest total research base, followed by cassava, while other crops received substantially less attention. There is pronounced geographic concentration in RSA research (Fig 5D). Asia led with 65 publications (44%), while Africa contributed only 14 (9.5%). This distribution resulted in a 4.6-fold difference between Asia and SSA.

### 3.7 RSA research: Geography and key methods

**Fig 6:**
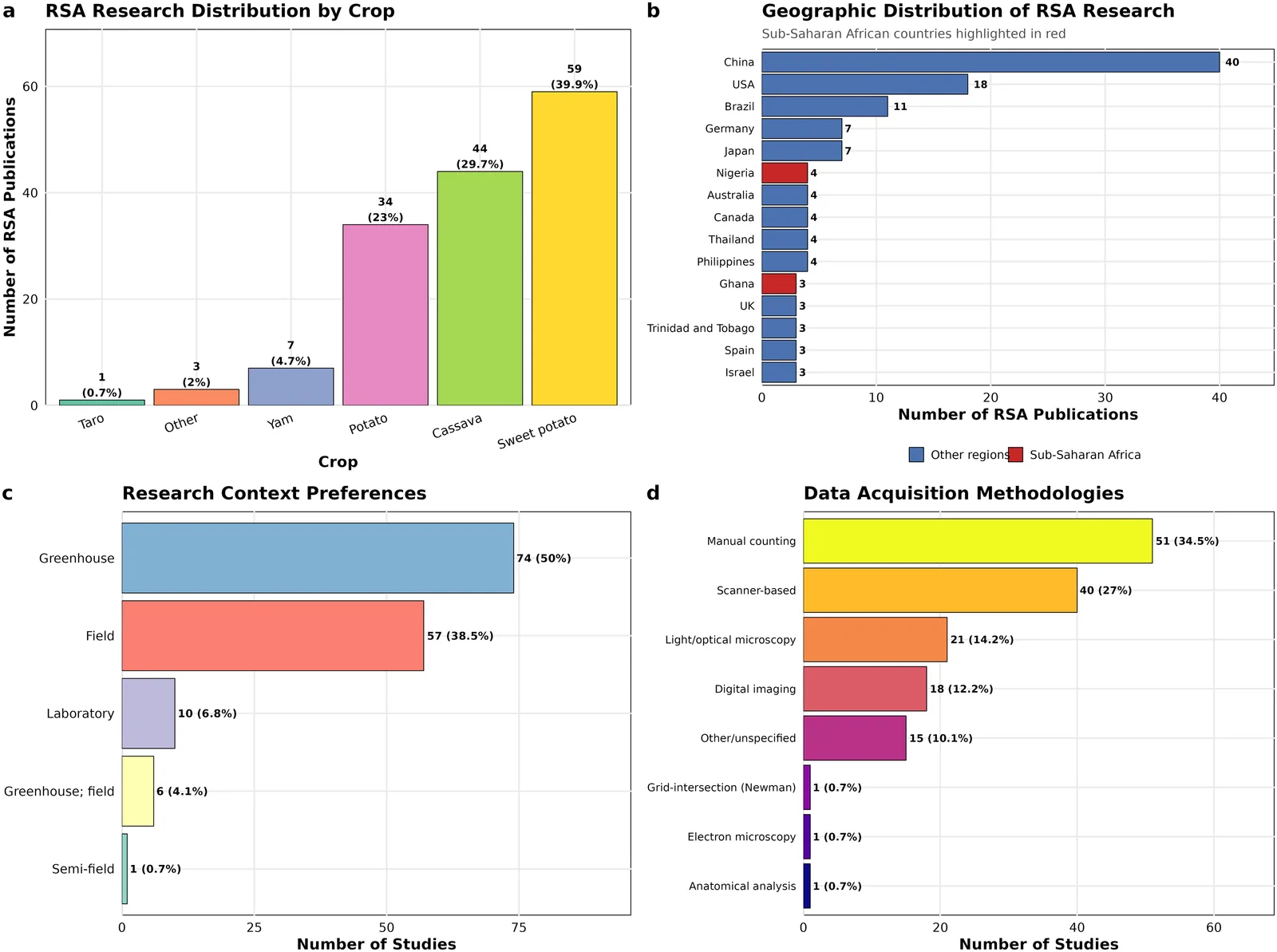
Distribution and methods of RSA research (n = 148). (A) Publications by crop (counts and percentages). (B) Publications by country, with sub-Saharan Africa highlighted. (C) Research context (greenhouse, field, laboratory, hybrid). (D) Data-acquisition methods (counts and percentages); studies reporting more than one method were assigned to their most advanced technique.

RSA research distribution across crops showed sweet potato’s dominance, with 56 publications (38%), followed by cassava with 46 publications (31%) and potato with 34 publications (23%) (Fig 6A). Seven publications addressed yams (5%) and one addressed taro (<1%); four further records (3%) concerned non-target crops. This distribution represents a 56-fold difference between the most- and least-studied crops in absolute RSA research output, sweet potato (56 publications) versus taro (a single study); cocoyam, with no recorded studies, is excluded from the ratio. China is the leading contributor, with 40 RSA publications, followed by the USA with 18 and Brazil with 11 (Fig 6B). Sub-Saharan African countries contributed 14 publications, representing 9.5% of the RSA research output, led by Nigeria (4) and Ghana (3). Greenhouse work dominated with 74 studies (50%), followed by field studies with 57 publications (39%) (Fig 6C). Laboratory studies accounted for 10 publications (7%), while greenhouse-field combinations contributed six studies (4%). Manual counting was the predominant approach with 51 studies (34.5%), followed by scanner-based analysis with 40 studies (27.0%) (Fig 6D). Light or optical microscopy accounted for 21 studies (14.2%) and digital imaging for 18 studies (12.2%), with a further 15 studies (10.1%) using other or unspecified methods. Advanced techniques were rare: electron microscopy, anatomical analysis, and the grid-intersection (Newman) method each appeared in a single study (0.7%).

### 3.8 RSA temporal evolution, geographic-methodological patterns, and trait category

**Fig 7:**
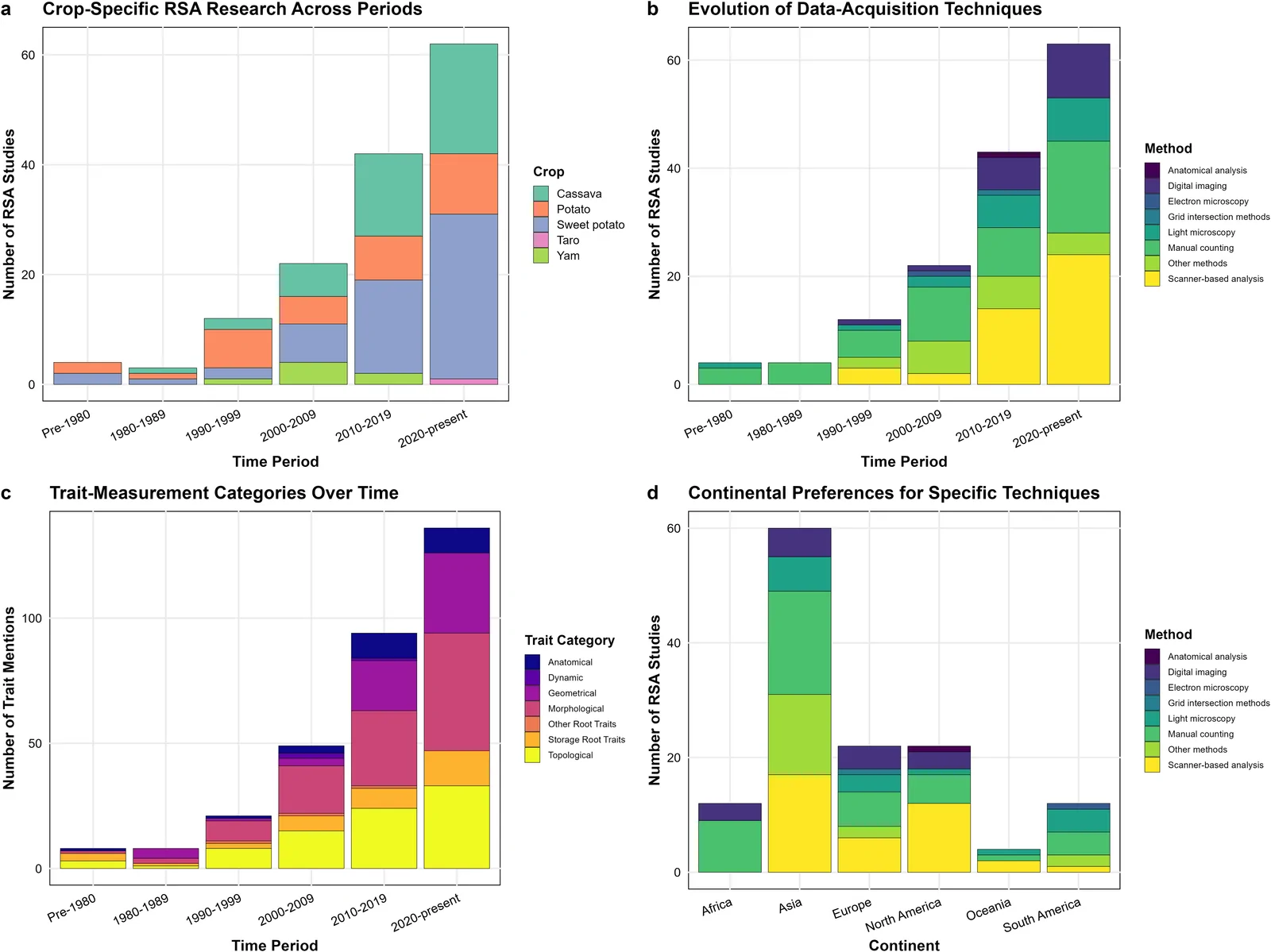
Temporal and geographic-methodological patterns in RSA research. (A) Crop-specific research across periods. (B) Evolution of data-acquisition techniques. (C) Trait-measurement categories over time. (D) Continental preferences for specific techniques.

The literature suggested a progressive expansion of RSA research, from fewer than 5 studies in earlier periods to 60 studies in 2020-present (Fig 7A). Sweet potato research has been a dominant area of study, contributing approximately 32 studies from 2020 to the present. Cassava research has shown concentrated growth in recent decades, with approximately 18 studies published from 2020 to the present, compared with minimal representation in earlier periods. Potato studies remained relatively stable over time, with 8–12 studies per decade since 2000, while yam and taro research remained sporadic, with fewer than 5 studies per period. The methodological evolution assessment showed a distinct temporal pattern of adoption (Fig 7B). Early periods (pre-1980–1990–1999) relied exclusively on manual counting and other basic methods, with fewer than five total studies per period. Scanner-based analysis became a dominant method from 2020 to the present, with approximately 25 studies, while manual counting contributed approximately 20 studies in the same period. Digital imaging showed progressive adoption from 2000 onward, reaching approximately 8 studies in 2020-present. Light microscopy and other advanced methods remained limited across all periods, with fewer than five studies each.

RSA temporal-trait category patterns showed morphological traits dominating across all periods, reaching approximately 80 studies in 2020-present (Fig 7C). Topological traits demonstrated steady growth from fewer than 10 studies in early periods to approximately 50 studies in 2020-present. Geometrical traits contributed to approximately 30 studies in the most recent period, while dynamic, anatomical, and storage root traits each contributed to fewer than 10 studies across all periods. Asia dominated, with 65 RSA studies in total, primarily employing scanner-based analysis (approximately 30 studies) and manual counting (approximately 20 studies) (Fig 7D). North America contributed 23 studies. Europe accounted for 24 studies, while Africa accounted for 14, and South America contributed 12 studies.

### 3.9 Current research disparities and food security challenges

**Fig 8:**
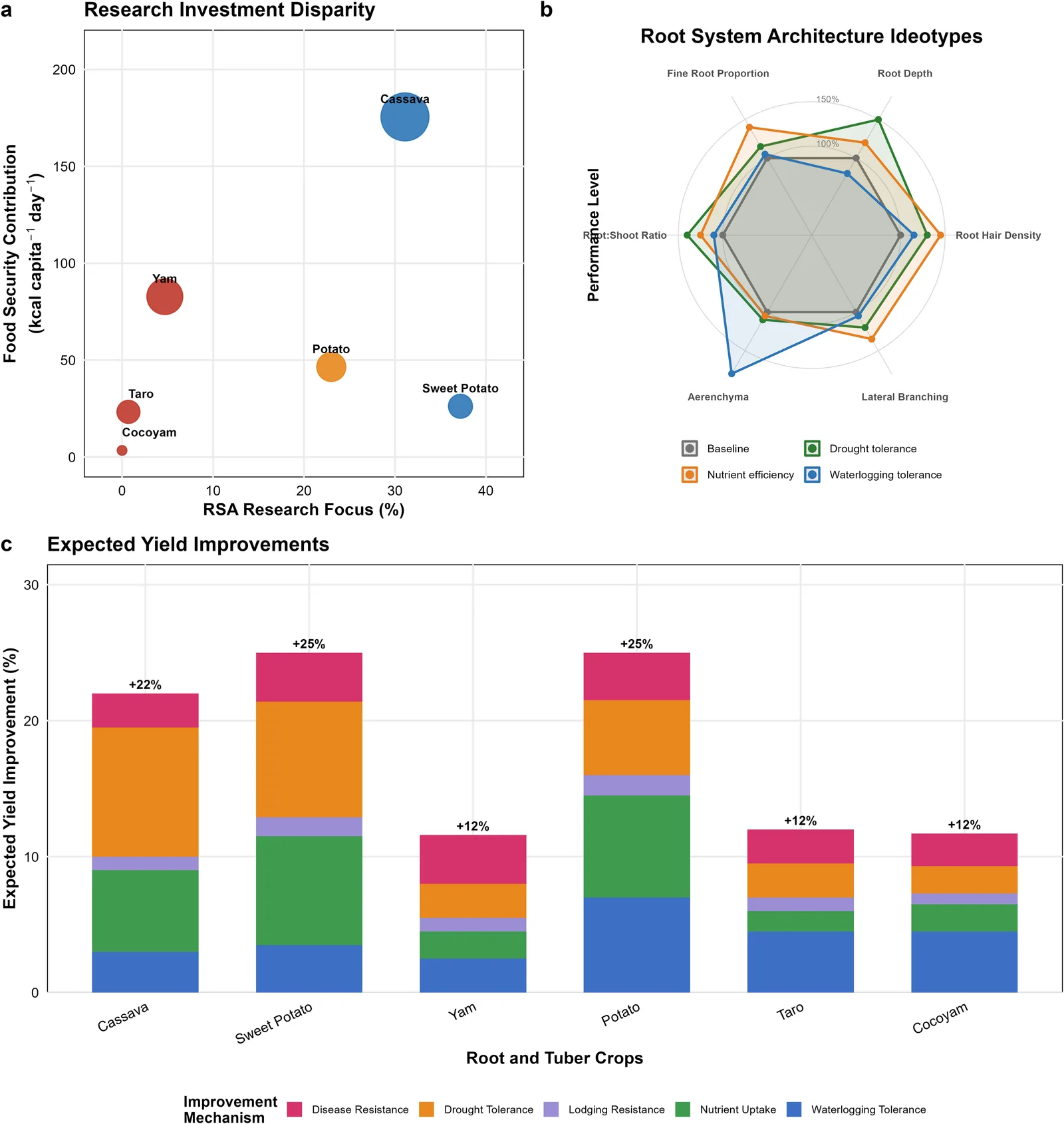
Problems and potential solutions in RSA research for sub-Saharan RTCs. (A) Research-investment disparity; bubble size = production volume, colour = research status (red = complete neglect). (B) Stress-specific RSA ideotypes, as a percentage of current performance (100% = baseline). (C) Expected yield improvements from RSA enhancement.

The bibliometric analysis showed disparities in research attention across crops (Fig 8A). Sweet potato dominates RSA research, accounting for 37.8% of publications, which is 1.2 times higher than cassava (31.1%) and 1.6 times higher than potato (23.0%), respectively. Yams receive limited attention, with only 4.7% of studies, representing an 8-fold deficit in research relative to sweet potatoes. Taro and cocoyam have received almost no documented research attention in RSA (Table 1).

### 3.10 Modelled RSA solutions and yield-improvement potential

The analyses in this subsection are model-based projections rather than empirical observations. Theoretical modelling of research investment disparity revealed a misalignment between caloric availability and RSA research attention, with cassava, sweet potato, and yam positioned in the high-importance, low-research-focus quadrant (65−85% food-security importance, 0.10–0.20 RSA proportion). In contrast, taro and cocoyam received no research attention despite measurable caloric availability (Fig 8A). Modelled stress-specific RSA ideotypes define distinct architectural requirements for optimal performance under different environmental constraints (Fig 8B). Drought tolerance ideotypes emphasise enhanced rooting depth (150% of current performance) and increased root-to-shoot ratios (140%). In comparison, nutrient-efficiency ideotypes prioritise root hair density (145%) and lateral root branching (135%) to improve soil exploration. The model suggested that the architectural improvements could translate into substantial yield improvement potential (Fig 8C). Sweet potato has the highest modelled improvement potential (25.4%), driven by its well-established research foundation in RSA. However, neglected crops show significant untapped potential: yams show an 11.6% improvement despite receiving only 4.7% of research attention. Taro and cocoyam show potential of 11.5% and 11.7%, respectively, but are severely constrained by a lack of research.

### 3.11 Investment analysis, economic returns, and research investment prioritisation matrix

**Fig 9:**
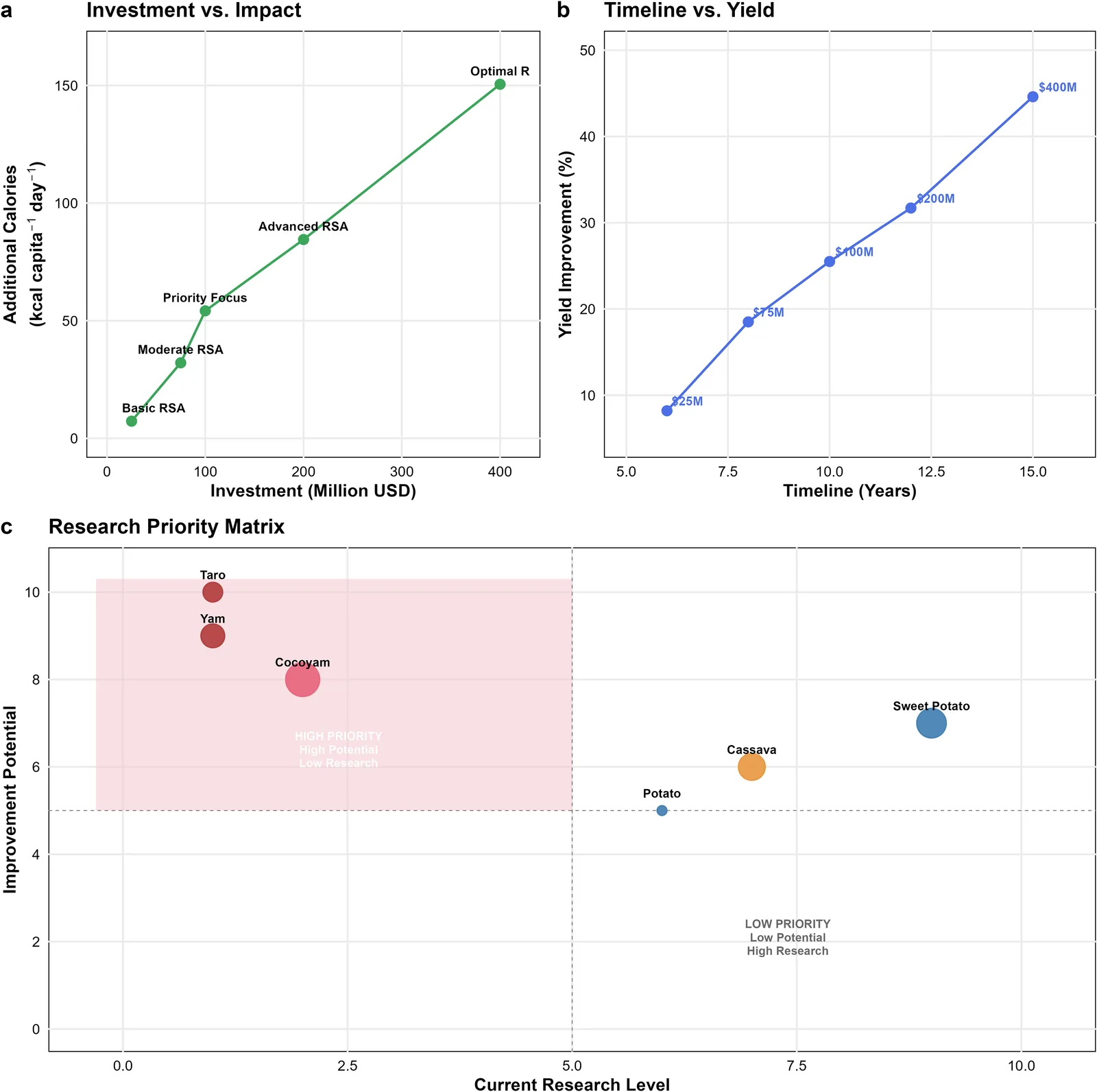
Investment Analysis and Pathway to Enhanced Food Security. (A) Investment vs impact relationship; (B) Timeline vs yield relationship; (C) RSA research-to-food security pathway.

Investment scenario modelling indicates positive relationships between research commitment and food security outcomes (Fig 9A, Table 2). The basic RSA scenario ($25M over 6 years) projects 7.3 additional kcal capita^-1^day^-1^, scaling to 150.5 kcal capita^-1^day^-1^ under optimal investment ($400M over 15 years) (Table 2). Fig 9B shows temporal requirements for achieving improvements. Within the model, longer investment periods lead to greater caloric gains. Strategic research prioritisation ranks crops according to current research levels relative to improvement potential, indicating investment priorities (Fig 9C). The HIGH PRIORITY quadrant identifies cocoyam and taro as research-investment-efficiency opportunities, with the highest modelled improvement-potential scores (9–10) and no current RSA research attention; cocoyam's placement is provisional, as it is absent from the production data and not reported separately in the food-supply data. These crops represent substantial knowledge gaps that the model prioritises for research attention, rather than crops with demonstrated economic returns. Yam has a high strategic priority, with moderate improvement potential (8) but severely limited current research (2); within the model, this implies high returns on enhanced investment. The 8-fold research deficit for sweet potatoes, combined with yams’ substantial importance for food security, establishes yams as a priority for immediate research investment. The LOW PRIORITY quadrant contains well-established crops (such as sweet potato and cassava) that, while important for continued advancement, offer diminishing returns compared to neglected alternatives. Under the model assumptions, investment-efficiency analysis implies a 3–5-fold advantage for neglected crops, with yam, taro, and cocoyam showing 8–15% improvement potential per million dollars of research investment, compared to 2–4% for well-researched crops. Return on investment analysis yields positive ratios across all scenarios (Table 2), with the priority-focus scenario at the highest modelled efficiency (~10:1 ROI) through targeted investment in neglected crops. The economic analysis indicates that moderate RSA investment (~8:1) and advanced RSA investment (~8:1) yield substantial modelled returns. The priority-focus scenario is the most efficient, concentrating resources on under-researched crops with the greatest potential for improvement. Social-impact projections indicate scaling of farmer beneficiaries across the investment scenarios (Table 2). The basic RSA scenario benefits 0.5 million farmers, progressing through moderate RSA (0.9 million) and advanced RSA (1.4 million) to optimal RSA (1.7 million). The priority-focus scenario combines substantial social impact (1.1 million farmers) with high modelled economic efficiency. These investment-scenario figures are model projections under the stated assumptions and are presented as scenario illustrations and optimistic upper bounds rather than expected outcomes.

**Table 2:** Investment-scenario analysis: projected impacts of different commitment levels to an RSA breeding programme. All scenarios yield modelled ROI ratios above 5:1 under the stated assumptions.

Scenario	Investment (M$)	Timeline (years)	Additional Calories (kcal capita^-1^ day^-1^)	ROI Ratio	Farmers Benefited (M)
Basic RSA	25	6	7.3	5.2:1	0.5
Moderate RSA	75	8	32.1	7.7:1	0.9
Advanced RSA	200	12	84.5	7.6:1	1.4
Optimal RSA	400	15	150.5	6.8:1	1.7
Priority Focus	100	10	54.2	9.8:1	1.1

## 4 Discussion

### 4.1 RSA research on RTCs: Global bibliometric overview

Root system architecture (RSA) plays important functional roles in crop adaptation and resilience to harsh or poor environmental conditions. With climate change and resource scarcity threatening to destabilise food security, even in advanced economies, interest in RSA research is growing rapidly. Given that root and tuber crops (RTCs) derive their edible parts from root systems and are the second largest source of dietary energy in the world, or the most important source of dietary energy for the poorest, it would be expected that RTCs would be strongly represented in the growing interest in RSA research, especially in regions that strongly depend on these crops for food security and livelihoods. Our bibliometric analysis suggests that although outputs may be increasing, RTCs have not received the desired or expected research attention for RSA compared with cereals. This neglect is even more pronounced in regions that depend strongly on RTCs. Temporal trends indicate that the proportion of RSA research should be interpreted with caution for the period before 2000, when fewer than 10 studies were available per period. The apparent peak RSA proportion in the 1980s (Fig 5B) reflects small absolute numbers, typically one to three studies per year, rather than any structured research priority. Meaningful temporal trends are interpretable from the year 2000 onwards, when publication volumes (>20 studies per period) provide sufficient representation to reflect genuine disciplinary patterns. From 2000 to the present, the RSA proportion declined gradually from approximately 25% to 18–20%, indicating that overall RTC research output has grown faster than RSA-specific output, a pattern consistent with the broader shift toward genomics, biochemistry, and food science applications in the post-2000 literature.

Our analysis points to severe disadvantages affecting crops designated as NUS and also receiving minimal RSA research attention (Fig 5C). Cassava, sweet potato, and yam cluster in the high food security importance, low RSA focus quadrant (0.10–0.20 RSA proportion, 65–85% food security importance), while potato occupies the inverse position with high RSA focus (0.32 proportion) but lower SSA food security relevance (45% importance). The African Orphan Crops Consortium (AOCC; https://africanorphancrops.org/meet-the-crops/) lists several RTCs, including yams, taro, and cocoyam, as NUS. Even when these crops receive some level of research attention, RSA research is often neglected, creating a compounding disadvantage: species already marginalised by international research priorities face additional neglect in RSA research (double neglect), precisely the research area that is arguably most critical for their improvement. This creates a profound irony in which crops whose economic value derives entirely from belowground organs receive minimal research attention on the very root systems that determine their productivity.

We also found an inverse relationship between the overall increase in RTC research output and RSA's proportional contribution over the study period. This indicates that although absolute output in RTC increased, root system research failed to maintain its proportional representation compared with other research areas (Fig 4C). This pattern suggests that institutional priorities might have shifted toward immediately applicable research areas such as genetics/molecular biology and biochemistry/physiology, potentially at the expense of fundamental root architectural studies that could provide long-term breeding targets.

Regarding general research on RTCs, there are significant regional, national, and crop variations in research output. Asia and North America dominate research output in RTCs for the period studied. Globally and in Asia, China leads research output on RTCs, with a focus on sweet potatoes (making sweet potatoes the most-studied RTC), followed by North America (the USA) on potatoes. This geographic distribution of RTC research outputs reflects differences in interest in, or capacity for, research to support agricultural modernisation, commercialisation, and domestic food security goals. Both the USA and China are global economic powerhouses. However, while the USA is a net food exporter, China is a net food importer, aiming to reduce its reliance on external supplies and maintain domestic agricultural capability. Africa, which relies heavily on cassava, contributed very little to RTC research output during the studied period. This might reflect a weak research capacity or a misalignment between the value of cassava for food security and livelihoods, on the one hand, and the investment of research resources and effort in the agricultural sector, on the other.

Specifically in RSA research, there are important variations in the tools and approaches used, as well as in the root traits or parameters studied. In China and Asia, which lead in RSA publications on RTCs, scanner-based analysis and manual counting methods are commonly used. In contrast, a range of methods and tools are used in North America. This might be due to the ease of use and high accessibility of scanner-based analysis for phenotypic studies. Thus, manual counting and scanner-based studies could be the most accessible, cost-effective, and practical tools for rapid RSA studies in resource-poor contexts. Moreover, the predominance of traditional manual counting reflects both the absence of high-throughput phenotyping capabilities and the inherent difficulties in studying RTC root systems (Fig 6D). Root and tuber crops pose unique phenotyping challenges that distinguish them from cereals, legumes, and other crop species. The dual functionality of root systems, with some roots serving traditional functions (anchorage, water, and nutrient acquisition) while others differentiate into storage organs, complicates distinguishing between root types and their respective contributions to plant performance. This functional differentiation means that conventional RSA metrics developed for fibrous or taproot systems may inadequately characterise the architecture of species in which root specialisation determines economic yield. It is noteworthy that the distinction between roots serving ‘traditional’ functions, anchorage, water and nutrient acquisition, and those that have differentiated into storage organs is a useful working framework, but recent work cautions against treating this as a rigid dichotomy. Gregory and Wojciechowski (2020) noted that some roots in RTCs may retain absorptive capacity even after differentiating into storage organs, raising the possibility that the functional boundary between these root types is more porous than currently assumed. Direct evidence comparing water and nutrient uptake capacities between storage and non-storage roots in cassava, sweet potato, or yam remains scarce. Gregory and Wojciechowski (2020) explicitly highlight this as a research priority, emphasising the need to characterise the longevity and functionality of different root types, particularly storage roots. The question is not merely academic; if storage roots do contribute meaningfully to soil resource acquisition, this would reshape ideotype design in RTCs, requiring trait targets that simultaneously optimise storage organ development and absorptive function. Resolving this requires dedicated root-system architectural, anatomical, physiological, and molecular characterisation of the functional capacity of storage roots across the development cycle, particularly under field conditions in sub-Saharan African agroecosystems, where soil resource constraints are most severe. The bulkiness and often irregular morphology of storage roots present additional technical challenges for high-throughput phenotyping systems designed for smaller, more uniform root systems or root crowns. The economic value of the root system creates a tension between destructive sampling required for detailed root analysis and the desire to preserve valuable plant material for yield assessment or breeding.

Beyond manual counting and camera-based imaging protocols, other methods and tools could impose logistical constraints in jurisdictions with weak infrastructure and limited research and development resources for RTCs. As expected, the emergence of scanner-based analysis after 2000 represents a critical technological transition, enabling higher-throughput phenotyping while maintaining accessibility for institutions with modest equipment budgets. The progressive adoption of digital imaging from 2000 onward demonstrates a gradual integration of more sophisticated approaches as computing power and imaging technology became more accessible (Fig 7B). This seems to have been the trajectory and strategy of Asian countries. However, the continued dominance of manual counting and scanner-based approaches indicates that research communities have prioritised reliability and cost-effectiveness over technological sophistication, a pragmatic approach given the resource constraints characterising much of global RTC research.

We found that studies focused mostly on morphological traits throughout the study period (Fig 7C). Of course, morphological traits are foundational to RSA studies and can be easily measured using the dominant methods and tools described earlier. They may also be easier to relate to growth and developmental processes in crop plants than other traits. Nonetheless, we found an increasing number of studies on topological traits over time, indicating a growing appreciation for and interest in how branching patterns and architectural complexity determine plant performance. However, limited attention to storage root traits represents a critical knowledge gap, as these traits determine economic yield in RTCs. This is the greatest mark of neglect in RSA research on RTCs, but there could be several reasons for this. For example, research focusing on absorptive or general architectural features rather than crop-specific storage organ characteristics may reflect the application of research methods or structures developed for fibrous root systems in cereals or legumes, rather than approaches optimised for crops in which root specialisation determines productivity [11]. Thus, further research should transcend the current dominance of morphological and topological (i.e., basic architectural and structural complexity) measurements, such as root diameters, lengths, orientations, numbers, branching patterns, and spatial relationships, which provide a foundational understanding of RSA.

The new research agenda should include due consideration of dynamic traits (growth rates and developmental processes) and anatomical traits (tissue-level characteristics) to deepen understanding of how root systems develop in response to environmental changes, which is critical knowledge for breeding programmes targeting stress tolerance. This could be facilitated by current technologies such as ground-penetrating radar, machine learning, and artificial intelligence-based phenotyping, as well as other non-destructive imaging techniques, for in situ monitoring of storage root development [16,44]. Furthermore, there appears to be a preference for RTC RSA studies conducted in a controlled environment (e.g., a greenhouse) rather than in the field. This could reflect both methodological convenience and the technical challenges associated with studying RTC root systems under realistic production conditions (Fig 6C). While controlled environments confer much control over growth conditions and enable precise measurement of individual traits, they fail to capture the complex soil-plant-climate interactions that determine RSA performance under field conditions.

### 4.2 Africa and RSA research on RTCs: Dependence and contributions

Sub-Saharan Africa continues to rely heavily on RTCs for food security and livelihoods, with consumption reaching 603.5 kcal/capita/day in Central Africa, accounting for 40.0% of total calories from major crops (Fig 1A, 1B). Apart from Central Africa, which showed the highest diversity (H’ = 0.780), the studied countries in Africa seem to have a concentrated dependence on one or a few RTCs. This signals vulnerability [45] and may create conditions for neglect (Fig 2D) [46]. Recent interest in the multi-use of RTCs in industrial, manufacturing, agricultural, and domestic settings may lead to increased expectations for investment in RSA research. Our results show that Africa's contribution to RSA research on RTCs is low. Candidate explanations, which require testing against funding and institutional data, include weak research infrastructure and investment, and a possible sidelining of RTCs in favour of cash crops and the so-called high-value crops (e.g., fruits and vegetables with short cycles). Persistent and rising food insecurity in Africa could partly arise from increasing reliance on imported cereals (fossil foods) [47] rather than RTCs that are suited to local ecological and production conditions, and predominantly produced by smallholder farmers using locally adapted varieties and traditional knowledge systems [1,3]. This structural dynamic, the prioritisation of exportable cash crops to secure foreign exchange, combined with growing dependence on imported cereals, and the consequent sidelining of locally important RTCs in both policy and research agendas, receives insufficient attention in mainstream food insecurity analyses. We propose it as one of the more important underlying drivers of the research neglect documented in this paper, a hypothesis that warrants testing with funding and political-economy data, and addressing it would require both additional resources and a reorientation of research and development priorities toward crops that sustain the food security of the rural poor.

Our findings suggest that relevant, context-specific knowledge of the RSA characteristics of RTCs in Africa, as well as their responses to environmental cues, is limited. Africa’s low contribution to global RTC research, particularly in RSA, creates a concerning research dependence (Fig 5D). Worse, RSA's low contributions came only from Nigeria and Ghana. Ghana’s three cassava-focused RSA studies from 2018–2020 [48–50] represent the entirety of documented RSA contributions on RTCs for the country. This signals an urgent need for increased investment and efforts in RTC RSA research, fit for supporting local production conditions [51]. No RSA work seems to have been done on taro and cocoyam, despite their nutritional contributions and high potential for improvement. Taro’s importance in wetland cultivation systems and cocoyam’s role in forest-agriculture interfaces represent unique production environments that could benefit substantially from RSA optimisation. Yet, these crops receive no documented research attention (Table 1). Similarly, yams’ cultural significance and production challenges in West Africa, where they serve as both a staple food and an important ceremonial crop, warrant the attention they deserve. Research neglect has rendered these important crops orphan or underutilised. We interpret this low share as reflecting where research is produced and funded, rather than the dietary importance of these crops, with publication access and indexing biases likely contributing.

### 4.3 Pathways to improvement and research prioritisation

Turning from the observed patterns to the modelled scenarios, the economic analysis suggests consistently positive returns, with ROI ratios ranging from ~5:1 (basic RSA) to ~10:1 (priority focus) (Table 2). These returns compare favourably with documented agricultural research investments, which typically yield a 4–9:1 ratio over 15–20-year periods (Alston et al., 2010; Hurley et al., 2014). In the model, the priority-focus scenario is the most efficient, concentrating resources on under-researched crops with the greatest potential for improvement, supporting the economic logic of investing in NUS. The progression from 7.3 additional kcal/capita/day (basic RSA) to 150.5 kcal/capita/day (optimal RSA) illustrates the scaling potential of coordinated research investment (Fig 9A). The timeline analysis, showing 8% improvements under 6-year scenarios versus 40% under 15-year programmes, indicates that larger modelled gains require sustained commitment (Fig 9, Fig 9B). We stress that these return-on-investment figures are projections from a deliberately simplified model rather than precise estimates. They rest on strong assumptions: a static population with no demographic projection, a simplified translation of yield gains into calories, stable farmgate prices, logistic adoption with no market saturation, and benefits persisting across a variety's lifespan, which together constitute the principal source of uncertainty in the economic analysis rather than a secondary limitation. The scenarios should therefore be read as indications or scenario illustrations of potential magnitude, not forecasts.

While these projections may represent optimistic upper bounds due to simplified assumptions about trait heritability, farmer adoption rates, and market dynamics, they illustrate the potential of dedicated RSA research in RTCs. Additional model limitations include limited data on RSA breeding success rates in RTCs, the exclusion of climate change impacts and temporal dynamics, and the failure to capture gene-by-environment interactions that may limit trait expression under field conditions. Even so, the theoretical framework provides guidance for research prioritisation and investment allocation, even if actual returns prove more modest. Historical precedents from cereal improvement programmes suggest that sustained investment in fundamental crop science can yield substantial productivity gains, with the Green Revolution demonstrating 1.4- to 3-fold yield increases through targeted breeding approaches [52,53]. The economic logic remains convincing from an efficiency perspective, even if realised benefits are only 50% of projected values; neglected crops would still offer marginal returns of 4:1–5:1 compared to 2.5:1–3:1 for incremental investment in well-studied systems, consistent with continued misallocation of research resources within the model. The modelled economic-efficiency analysis indicated marginal returns inversely related to baseline research intensity. This pattern reflects standard economic principles of diminishing marginal returns, where incremental investment in well-studied systems (cassava, sweet potato) yields lower marginal productivity than initial investment in research-deficient crops (taro, cocoyam, yam). From a portfolio optimisation perspective, efficient research allocation would shift resources toward currently neglected crops until marginal returns equalise across the portfolio. However, transaction costs, institutional path dependence, and capacity constraints may prevent such rational reallocation despite the efficiency gains implied by the model. The magnitude of projected food security returns under optimal allocation suggests current patterns represent substantial welfare losses for populations dependent on neglected crops, though realising these gains requires addressing the structural barriers to research investment identified in this analysis.

In the research prioritisation matrix, crops were grouped by current research investment versus improvement potential, identifying cocoyam and taro as high-priority targets occupying the maximum-efficiency quadrant (Fig 9C). These crops combine substantial modelled improvement potential with complete research neglect, marking them as priorities where the knowledge gap is largest rather than as crops with demonstrated economic returns; cocoyam's placement is the most tentative, as it lacks separate food-supply and production statistics (Table 1) and rests on its documented regional role rather than complete data. Realising the theoretical potential identified in this analysis requires coordinated investment in several key areas. First, African countries must prioritise the development of agricultural research infrastructure, including specialised facilities for RSA research. The establishment of regional plant phenomics centres dedicated to RTC research, similar to successful models such as the Plant Phenotyping and Imaging Research Centre (P2IRC) in Canada, the European Plant Phenotyping Network facilities in Jülich, Germany, or the Australian Plant Phenomics Facility, could provide the technical capabilities currently lacking across SSA institutions. Such centres could serve multiple countries, providing training opportunities, standardised methodologies, and high-throughput phenotyping capabilities that individual nations cannot afford on their own.

Second, capacity building initiatives must address the human resource constraints that limit research productivity. Brain drain continues to affect African agricultural research, with trained scientists migrating to institutions with better infrastructure and funding opportunities. Sustained investment in competitive salaries, research support, and career development pathways could help retain talent while attracting diaspora scientists back to regional institutions. Third, international partnerships must be restructured to ensure that research priorities align with African food security needs rather than donor-country interests. Collaborative networks linking SSA institutions with international centres should emphasise South-South cooperation and technology transfer, building regional expertise rather than perpetuating dependence on external technical assistance.

Fourth, investment in advanced phenotyping technologies specifically adapted to RTCs is essential. This includes developing non-destructive imaging techniques that can handle the irregular morphology and large size of storage roots, creating protocols for distinguishing between functional root types, and establishing standardised trait names and measurement procedures across different RTC species. The research-to-food security pathway provides a conceptual framework for translating RSA investments into enhanced regional nutrition (Fig 9C). This progression from current limited knowledge through enhanced ideotypes, improved varieties, and widespread adoption to enhanced food security represents a logical sequence that requires coordinated effort across multiple disciplines and institutional contexts.

Once the infrastructure and human resource requirements have been met, a critical priority for advancing RTC productivity in SSA is establishing dedicated RSA breeding programmes for key RTCs. To date, RSA traits have not been fully incorporated into RTC breeding, with recognition of their potential to develop climate-resilient varieties a recent phenomenon [10]. In potatoes, the importance of measuring RSA response to drought has only recently been considered, whereas in cassava, variability in RSA response to soil conditions remains poorly characterised for breeding applications [10]. Future research, for example, can prioritise understanding of lateral root initiation, emergence, and development in RTCs, using advances in cereal crops as templates for identifying research goals [10, 54]. In sweet potato, successful lateral root development is associated with reduced lignification in adjacent stelar tissue and developmental competency mechanisms that require further elucidation for breeding applications [10]. Developing high-throughput phenotyping protocols specifically adapted to RTC root systems, establishing genetic mapping populations for RSA traits, and identifying molecular markers associated with beneficial RSA features could be essential for incorporating RSA into breeding programmes [8,55]. The integration of RSA breeding with storage root quality traits could unlock synergistic improvements in both productivity and nutritional targets under challenging growing conditions.

### 4.4 Implications for research priorities and policy

#### 4.4.1 Priority research gaps.

Our results suggest specific research gaps that constrain productivity improvements in RTCs important to African food security. Root system research on cassava, despite the crop's nutritional dominance, focuses on basic morphological characterisation rather than on traits that affect storage organ development and stress tolerance under field conditions. Yam research shows larger gaps, with minimal understanding of how root architecture influences tuber formation or of responses to soil fertility constraints typical in smallholder systems. Taro and cocoyam have received almost no systematic investigation of their root characteristics. These knowledge deficits translate directly to breeding programme limitations. Without understanding genotypic variation in root traits, selection for improved water capture during drought, enhanced nutrient acquisition in depleted soils, or waterlogging tolerance in low-lying areas remains empirically unsupported. Field-based research under realistic farming conditions is particularly sparse, with most studies employing controlled environments that may not reflect root behaviour in compacted, nutrient-poor, variable-moisture soils typical of African smallholder farms.

Methodological gaps compound these knowledge limitations. Root system phenotyping in RTCs presents distinct challenges that may be absent in cereals or legumes, for which most high-throughput phenotyping infrastructure has been developed. Cereals produce fibrous or crown root systems that primarily serve resource-acquisition functions, while legumes develop taproot architectures. RTCs, by contrast, develop functionally complex root systems in which individual roots may serve dual purposes. Some RTC roots function exclusively in water and nutrient uptake; others transform into storage organs that constitute the economic yield, and additional adventitious roots may emerge from developing tubers. Existing phenotyping technologies designed for cereal or legume root systems may not adequately characterise the architectural and functional complexities of these root systems. Distinguishing storage root initiation from non-storage lateral root development, tracking tuber expansion dynamics, and measuring root function partitioning between absorption and storage may require specialised imaging systems, temporal monitoring protocols, and analytical processes not available in standard phenotyping facilities. African research institutions lack access to these specialised systems. Non-destructive measurement technologies that enable temporal or repeated observations of the same plants throughout their development could accelerate understanding of root-to-tuber transformation processes, but such infrastructure is concentrated in high-income countries, where RTCs contribute minimally to food systems and research priorities.

Genomic resources for RTCs show substantial variation in development and utilisation. Genome sequences for several yam species became available between 2015 and 2020 [56–58], while taro genome assemblies completed in 2020 identified disease-resistance QTLs and generated molecular markers for breeding programmes [59,60]. Cassava genome assemblies [61,62] have enabled genome-wide association studies identifying loci for cassava mosaic disease resistance, cassava brown streak disease resistance, and yield traits, providing candidate genes and SNP markers for marker-assisted breeding [63–65]. However, translation of these genomic advances to improved cultivars remains constrained by limited research funding and inadequate international cooperation [66]. Cocoyam genomic research lags substantially behind other RTCs, with no complete genome sequence available for *Xanthosoma sagittifolium* despite efforts in molecular marker development and genetic linkage mapping [67]. Integration of root research with agronomic management represents another critical gap, as understanding interactions between cultivation practices and root characteristics could yield productivity gains that would otherwise be unattainable without genetic improvement.

#### 4.4.2 Research funding allocation mechanisms.

International agricultural research funders must implement explicit criteria to weight crop research priorities by demonstrated food security impact. CGIAR centres appear to allocate research resources primarily on the basis of historical precedent and existing infrastructure capacity rather than on systematic assessment of where marginal investment yields the greatest nutritional returns [21]. The current system tends to reward crops with established breeding programmes and molecular toolkits while creating entry barriers for neglected species lacking these foundations. A transparent prioritisation framework incorporating metrics such as the RCDI developed here would enable more rational resource allocation. National agricultural research systems in cassava-dependent countries allocate budgets across crops based on political economy considerations that often favour export commodities over staple-food-security crops. Ghana's institutional architecture illustrates these distortions. Cocoa, an export commodity, benefits from dedicated research infrastructure through the Cocoa Research Institute of Ghana (CRIG), which is backed by the Ghana Cocoa Board, whose substantial revenue streams come from export levies. CRIG employs over 200 researchers working on cocoa, shea, kola nut, and cashew. The Council for Scientific and Industrial Research (CSIR) operates the Oil Palm Research Institute (OPRI) with a similar dedicated focus on oil palm and coconut. Root and tuber crops, by contrast, fall within the general mandate of the CSIR’s Crops Research Institute (CRI) alongside cereals, legumes, and vegetables, competing for limited resources without dedicated institutional backing or earmarked funding. Cassava provides more calories to Ghanaians than cocoa does, and export revenue contributes to GDP, yet it receives a fraction of the research investment. Policy reforms establishing commodity boards or dedicated research mandates for food security crops, modelled on the COCOBOD structure, would create institutional mechanisms to sustain research investment in crops that sustain domestic nutrition rather than foreign exchange earnings. Our modelled investment-scenario analysis suggests that research targeting currently neglected crops offers higher marginal returns than incremental investment in well-studied systems. The modelled 10:1 return ratio for priority crops (taro, cocoyam, yam) compared to 5–6:1 for well-researched crops (cassava, sweet potato) suggests efficiency gains from strategic reallocation. Alston et al. [42] suggested that agricultural research investments in data-poor environments carry higher variance but offer substantial returns when successful. The policy challenge lies in creating funding mechanisms that accept this variance while maintaining accountability.

#### 4.4.3 Institutional barriers and governance.

The persistence of research neglect despite clear food security rationale is more consistent with institutional than with informational failures. Three candidate explanations, offered as hypotheses consistent with the literature rather than demonstrated causes, warrant attention. First, donor-driven research agendas may prioritise crops with market potential in donor countries. Bilateral research funding patterns across crops remain poorly documented, though available evidence suggests potential misalignment with recipient country priorities. For example, one European-funded agricultural research initiative in West Africa allocated resources to pineapple and mango research targeting export markets, while declining support for okra and eggplant breeding despite their importance in domestic food systems. Our bibliometric analysis reveals similar patterns at scale: potato receives 22% of research attention on root crops globally despite providing only 13% of RTC calories in sub-Saharan Africa, while yams provide 23% of calories but receive just 4% of research effort. The geographic concentration compounds this mismatch: Asia and North America account for 64% of root research publications, while sub-Saharan Africa accounts for 11%, despite hosting the majority of cassava production and nearly all yam cultivation. A systematic assessment of bilateral agricultural research funding by crop category and end-use would determine whether development assistance effectively targets food security priorities or inadvertently reinforces export-oriented agriculture. Second, infrastructure investments may follow path dependence. Research institutions in sub-Saharan Africa allocated resources unevenly across root and tuber crops during formative decades. Cassava research expanded substantially in the 1970s and 1980s, with the International Institute of Tropical Agriculture (IITA) in Nigeria devoting considerable capacity to cassava improvement since its establishment in 1967 [68]. Yams, taro, and cocoyams received research attention at IITA and other institutions, including research on disease resistance to white yam scorch and cocoyam root rot blight [69]. However, resource allocation remained disproportionate to economic importance. Igbozurike et al. [70] documented that yams, despite contributing $840 million to Nigeria’s economy in 1963, received far less research support than export commodities. This pattern of relative neglect or minimal institutional support, rather than an absolute absence of research, established capacity imbalances that persist into the current decades. Reversing these patterns requires deliberate capacity building in neglected crop systems. Third, prestige structures in agricultural science may create incentives favouring cereals over root crops. Publication opportunities, citation networks, and professional advancement pathways concentrate on cereal genetics and physiology.

Policy instruments addressing these barriers include: competitive grant programmes with explicit set-asides for neglected crop research; regional research centre mandates requiring minimum investment shares in crops other than cassava and potato; and bilateral aid requirements that recipient countries justify research portfolios against domestic nutritional priorities rather than export potential. The African Union's Comprehensive Africa Agriculture Development Programme provides a policy vehicle for implementing such reforms across multiple countries simultaneously.

#### 4.4.4 Implementation pathways and capacity constraints.

Translating research investment into productivity gains requires addressing capacity constraints that limit research execution in sub-Saharan Africa. The region contributes 9.5% of global root system research despite hosting 60% of cassava production and the majority of yam cultivation. This reflects genuine resource limitations rather than a lack of scientific interest. Equipment for root phenotyping (e.g., high-throughput root phenotyping systems, minirhizotron systems, ground-penetrating radar) costs $50,000-$200,000 per installation, which is beyond the capital budgets of most African universities. Technical expertise in root trait measurement and genetic mapping remains concentrated in institutions in North America, Europe, and Asia.

Three implementation approaches merit consideration. The hub-and-spoke model would establish 3–4 regional root phenotyping centres serving multiple countries. The CGIAR system's existing research stations in Nigeria (IITA), Kenya (multiple centres), Zimbabwe, and universities provide potential sites. Capital investments of $10–20 million can equip these facilities with field phenotyping infrastructure and imaging systems. National programmes could send materials and personnel for analysis while building local capacity. The graduate training pathway would expand PhD programmes in root biology at African universities. The current annual production of root biology or root phenomics PhDs across sub-Saharan Africa is probably very low compared with other disciplines. Targeted fellowship programmes could increase this within five years. The South-South collaboration approach would formalise partnerships between Asian institutions with established root crop research programmes (India, Indonesia, Philippines) and African counterparts. Technology transfer agreements and joint degree programmes would accelerate knowledge flows.

#### 4.4.5 Seed system policy and variety release.

Research that produces improved varieties has a food security impact only when farmers adopt them. Formal seed systems for root and tuber crops remain underdeveloped across sub-Saharan Africa. Cassava and yams propagate vegetatively, requiring different multiplication and distribution systems than cereals. Most countries lack certified seed programmes for crops other than cassava. Taro and cocoyam operate almost entirely through informal farmer-to-farmer exchange. Policy attention to variety release procedures, seed certification standards adapted to vegetatively propagated crops, and distribution networks would prevent research outputs from remaining confined to experimental stations.

Extension service capacity presents another constraint. Agricultural advisors receive training focused on cereal production practices. Root crop agronomy, pest management, and variety recommendations get minimal coverage in extension curricula. Updating training programmes and developing decision support tools for root crop cultivation would improve the translation of research into practice. Mobile phone-based advisory services piloted for cereals in East Africa [71–74], for example, could extend to root crop systems at modest marginal cost.

#### 4.4.6 Political economy of research neglect.

The prioritisation of export cash crops over subsistence food crops may reflect historical patterns established during colonial administration and reinforced by post-independence development strategies that emphasise foreign exchange generation. Countries expanded research on cocoa, coffee, tea, and cotton, while treating root crops as traditional subsistence commodities that required no scientific improvement [19,75–77]. This created research capacity imbalances that persisted for decades. Ghana maintains established research programmes in cocoa but has limited research programmes for yams and cocoyams, despite their higher domestic consumption. Policy discussions about agricultural research rarely explicitly examine these structural biases.

International development agencies may have contributed to this pattern by funding projects that favoured crops with export potential. Although the pattern shifted and cassava breeding in the 20^th^ century transformed it “from a poor man’s crop to an urban food” through long-term research efforts [78], Green Revolution investments concentrated on wheat and rice, bypassing root crops entirely. More recent initiatives promoting climate-smart agriculture and nutrition-sensitive farming provide opportunities to refocus on neglected food-security crops. Making these historical patterns explicit in policy discourse could build support for deliberate rebalancing of research portfolios.

### 4.5 Limitations

There are some limitations of this study that should be highlighted. The investment-return model is a scenario exercise rather than an empirical result: its yield, calorie, return-on-investment and beneficiary figures depend on strong assumptions, the direct translation of yield gains into calories, stable farmgate prices, logistic adoption, and trait multipliers drawn largely from cereal and legume evidence rather than measured in root and tuber crops, and are best read as optimistic upper bounds; a one-way sensitivity analysis (S2 Table) shows that the priority and moderate scenarios remain near 4–5:1 even when benefits are halved. The bibliometric data were limited to English-language records from Scopus and Google Scholar; agriculture-specific databases such as CAB Abstracts and AGRIS, and Web of Science, were not searched, so records indexed only in those sources may have been missed, and Francophone and Lusophone African literature is likely under-represented, though the deposited dataset allows the search to be extended. The Google Scholar search relied on a relevance-based stopping rule; screening was halted after results ceased to return relevant records, at 529 records, a necessarily subjective cutoff, though the deposited dataset allows the screened records to be inspected directly. Cocoyam and taro are not reported separately in the FAOSTAT food-supply data (they fall under “Roots, Other”), and cocoyam is absent from the production data, so the cocoyam ranking relies on its documented regional role rather than on complete statistics. Regional figures are reported as both unweighted and population-weighted country means (S3 Table); we treat the unweighted values as primary because the country is the unit of policy interest, while noting that population-weighting raises the estimates, so the unweighted figures are the conservative ones. Screening was carried out by two researchers with adjudication but without a computed inter-rater statistic. The Root Crop Dependency Index is a transparent descriptive index developed for this study rather than a formally validated instrument, and its robustness to the classification threshold is shown in S1 Table.

## 5 Conclusions

Agricultural research priorities in sub-Saharan Africa may be misaligned with the region's food-security needs. We interpret this misalignment as reflecting institutional path dependence and donor preferences rather than a systematic assessment of those needs, an interpretation that remains to be tested. Root and tuber crops supply 354 kcal/capita/day, over 40% of crop calories in several countries, yet research capacity is concentrated in high-income countries (Asia and North America account for 64% of publications) and on crops of minimal regional importance (potato attracts 23% of research attention while supplying 13% of calories). Sub-Saharan Africa, which hosts most production, contributes 11% of global research output of root and tuber crops. Within this imbalance, three crops face a double neglect: yam, taro and cocoyam receive between 0% and 4.7% of RSA research attention despite their documented nutritional importance in West and Central Africa.

Our investment-scenario modelling suggests that yield gains of 12–25%, with return ratios of 5:1–10:1, are achievable through strategic reallocation toward these neglected crops, returns that exceed historical averages for well-funded commodity research. These are modelled illustrations under stated assumptions, not demonstrated outcomes, and the central task now is to convert this potential into field evidence. Doing so will require funders and national research systems to weight crop research by demonstrated food-security impact rather than by infrastructure or market potential, on the model already applied to export commodities such as cocoa.

A misalignment between research attention and food-security need on this scale is both systematic and consequential. On this evidence, we prioritise three concrete actions: (1) targeted RSA phenotyping and breeding across the region’s root and tuber crops, cassava included, given that its research attention remains low relative to its caloric contribution, with priority to yam, taro and cocoyam, the crops of greatest food-security importance and least current attention; (2) building RSA datasets and phenotyping capacity within African research institutions; and (3) field validation of the modelled trait–yield relationships in root and tuber crops under smallholder conditions.

## Supporting information

S1 Fig PRISMA 2020 flow diagram of the literature search and screening process. Database searches of Scopus (n = 1,548; 17 and 21 April 2025) and Google Scholar (n = 529; 21 April 2025) returned 2,077 records. After removal of 920 duplicates, 1,157 records were screened, 1,004 were assessed for eligibility, and 893 publications met the inclusion criteria and formed the bibliometric dataset; of these, 148 root system architecture–focused studies were analysed in detail.
(TIF)

S1 Table Number and membership of high-dependency countries across RCDI thresholds (country-level means, 2018–2022). Robustness of the high-dependency classification to the choice of RCDI cut-off.
(DOCX)

S2 Table Illustrative one-way sensitivity of ROI to benefit-side assumptions. “Combined” corresponds to roughly a 50% reduction in realised benefits.
(DOCX)

S3 Table Mean root-and-tuber caloric supply (kcal capita^-1^ day^-1^) by region, unweighted versus population-weighted. Population weights are World Bank World Development Indicators total population, 2020 midyear estimates.
(DOCX)

S1 Dataset Underlying data. Bibliometric dataset (893 publications), Root Crop Dependency Index by country, FAOSTAT food-supply and production data, and the population weights used for the weighted regional estimates.
(XLSX)

## References

[1] Scott GJ, Rosegrant MW, Ringler C. Roots and tubers for the 21st century: trends, projections, and policy options. Intl Food Policy Res Inst. 2000.

[2] Jennings D. Tropical root and tuber crops. Cassava, sweet potato, yams and aroids. Experimental Agriculture. 2009;45(3):382. doi: 10.1017/S0014479709007832

[3] Lebot V. Tropical Root and Tuber Crops. Cabi; 2019.

[4] Prain G, Naziri D. The role of root and tuber crops in strengthening agri‐food system resilience in asia: a literature review and selective stakeholder assessment. International Potato Center; 2020. doi: 10.4160/9789290605393

[5] Kumar JS, Sunitha S, Giri N. A review of tropical root and tuber crops for livelihood security and nutrition. Environment and Ecology. 2024;42(4A):1793–800. doi: 10.60151/envec/ftoz8778

[6] Rogers ED, Benfey PN. Regulation of plant root system architecture: implications for crop advancement. Curr Opin Biotechnol. 2015;32:93–8. doi: 10.1016/j.copbio.2014.11.015 25448235

[7] Lynch JP. Harnessing root architecture to address global challenges. Plant J. 2022;109(2):415–31. doi: 10.1111/tpj.15560 34724260 PMC9299910

[8] Khan MA, Gemenet DC, Villordon A. Root system architecture and abiotic stress tolerance: current knowledge in root and tuber crops. Front Plant Sci. 2016;7:1584. doi: 10.3389/fpls.2016.01584 27847508 PMC5088196

[9] Lynch JP, Mooney SJ, Strock CF, Schneider HM. Future roots for future soils. Plant Cell Environ. 2022;45(3):620–36. doi: 10.1111/pce.14213 34725839 PMC9299599

[10] Villordon AQ, Ginzberg I, Firon N. Root architecture and root and tuber crop productivity. Trends Plant Sci. 2014;19(7):419–25. doi: 10.1016/j.tplants.2014.02.002 24630073

[11] Gregory PJ, Wojciechowski T. Root systems of major tropical root and tuber crops: root architecture, size, and growth and initiation of storage organs. In: Sparks DL, editor. Advances in Agronomy. Academic Press; 2020. p. 1–25. doi: 10.1016/bs.agron.2020.01.001

[12] Zhu J, Ingram PA, Benfey PN, Elich T. From lab to field, new approaches to phenotyping root system architecture. Curr Opin Plant Biol. 2011;14(3):310–7. doi: 10.1016/j.pbi.2011.03.020 21530367

[13] Adu MO, Chatot A, Wiesel L, Bennett MJ, Broadley MR, White PJ, et al. A scanner system for high-resolution quantification of variation in root growth dynamics of Brassica rapa genotypes. J Exp Bot. 2014;65(8):2039–48. doi: 10.1093/jxb/eru048 24604732 PMC3991737

[14] Downie HF, Adu MO, Schmidt S, Otten W, Dupuy LX, White PJ, et al. Challenges and opportunities for quantifying roots and rhizosphere interactions through imaging and image analysis. Plant Cell Environ. 2015;38(7):1213–32. doi: 10.1111/pce.12448 25211059

[15] Piñeros MA, Larson BG, Shaff JE, Schneider DJ, Falcão AX, Yuan L, et al. Evolving technologies for growing, imaging and analyzing 3D root system architecture of crop plants. J Integr Plant Biol. 2016;58(3):230–41. doi: 10.1111/jipb.12456 26683583

[16] Joseph Fernando EA, Selvaraj MG, Delgado A, Rabbi I, Kulakow P. Frontline remote sensing tool to locate hidden traits in root and tuber crops. Mol Plant. 2022;15(10):1500–2. doi: 10.1016/j.molp.2022.08.010 36045578

[17] Weihs BJ, Heuschele D-J, Tang Z, York LM, Zhang Z, Xu Z. The state of the art in root system architecture image analysis using artificial intelligence: a review. Plant Phenomics. 2024;6:0178. doi: 10.34133/plantphenomics.0178 38711621 PMC11070851

[18] Nanbol KK, Namo O. The contribution of root and tuber crops to food security: a review. J Agric Sci Technol B. 2019;9(10):2161–6264.

[19] Edem ID, Nkereuwem ME. Crucial roles of tuber crops and the development activities in the global food system. Journal of Agricultural Science. 2015;2(2):42–9.

[20] Andrzejczak K, Przysiecka L. Agricultural Biotechnology Research in Sub-Saharan Africa. In: Managing Intellectual Capital and Innovation for Sustainable and Inclusive Society: Managing Intellectual Capital and Innovation; Proceedings of the MakeLearn and TIIM Joint International Conference 2015. 2015. p. 1031.

[21] Beintema NM, Stads GJ. A comprehensive overview of investments and human resource capacity in African agricultural research. 2017. https://hdl.handle.net/10568/146174

[22] Tadele Z, Bartels D. Promoting orphan crops research and development. Planta. 2019;250(3):675–6. doi: 10.1007/s00425-019-03235-x 31280328

[23] Fabbri A, Lai A, Grundy Q, Bero LA. The influence of industry sponsorship on the research agenda: a scoping review. Am J Public Health. 2018;108(11):e9–16. doi: 10.2105/AJPH.2018.304677 30252531 PMC6187765

[24] Pingali P, Raney T. From the green revolution to the gene revolution: how will the poor fare?. Development Economics between Markets and Institutions. Wageningen Academic; 2007. p. 407–22.

[25] Rivera E, S S, J VC. Distribution of pigeon peas, cassava, coffee and grass roots in an Ultisol [Coffea arabica, Manihot esculenta, Cajanus cajan, implications for crop management, Puerto Rico]. Journal of Agriculture of the University of Puerto Rico. 1983;67.

[26] Q. Subere JO, Bolatete D, Bergantin R, Pardales A, Belmonte JJ, Mariscal A, et al. Genotypic variation in responses of Cassava (Manihot esculentaCrantz) to drought and rewatering: root system development. Plant Production Science. 2009;12(4):462–74. doi: 10.1626/pps.12.462

[27] El-Sharkawy MA. Physiological characteristics of cassava tolerance to prolonged drought in the tropics: implications for breeding cultivars adapted to seasonally dry and semiarid environments. Braz J Plant Physiol. 2007;19(4):257–86. doi: 10.1590/s1677-04202007000400003

[28] Ahmadi J, Pour-Aboughadareh A, Fabriki-Ourang S, Mehrabi A-A, Siddique KH. Screening wheat germplasm for seedling root architectural traits under contrasting water regimes: potential sources of variability for drought adaptation. Archives of Agronomy and Soil Science. 2018;64(10):1351–65. doi: 10.1080/03650340.2018.1432855

[29] Mathew I, Shimelis H, Mutema M, Clulow A, Zengeni R, Mbava N, et al. Selection of wheat genotypes for biomass allocation to improve drought tolerance and carbon sequestration into soils. J Agronomy Crop Science. 2019;205(4):385–400. doi: 10.1111/jac.12332

[30] Wang X, Yan X, Liao H. Genetic improvement for phosphorus efficiency in soybean: a radical approach. Ann Bot. 2010;106(1):215–22. doi: 10.1093/aob/mcq029 20228090 PMC2889788

[31] Vandamme E, Renkens M, Pypers P, Smolders E, Vanlauwe B, Merckx R. Root hairs explain P uptake efficiency of soybean genotypes grown in a P-deficient Ferralsol. Plant Soil. 2013;369(1–2):269–82. doi: 10.1007/s11104-012-1571-2

[32] Nestler J, Wissuwa M. Superior root hair formation confers root efficiency in some, but not all, rice genotypes upon P deficiency. Front Plant Sci. 2016;7:1935. doi: 10.3389/fpls.2016.01935 28066487 PMC5174101

[33] Zhu and Lynch. The contribution of lateral rooting to phosphorus acquisition efficiency in maize (Zea mays) seedlings. 2004. Accessed 22 October 2010. http://cat.inist.fr/?aModele=afficheN&cpsidt=16271710 10.1071/FP04046 32688963

[34] Jia X, Liu P, Lynch JP. Greater lateral root branching density in maize improves phosphorus acquisition from low phosphorus soil. J Exp Bot. 2018;69(20):4961–70. doi: 10.1093/jxb/ery252 30295904 PMC6137997

[35] Abiko T, Kotula L, Shiono K, Malik AI, Colmer TD, Nakazono M. Enhanced formation of aerenchyma and induction of a barrier to radial oxygen loss in adventitious roots of Zea nicaraguensis contribute to its waterlogging tolerance as compared with maize (Zea mays ssp. mays). Plant Cell Environ. 2012;35(9):1618–30. doi: 10.1111/j.1365-3040.2012.02513.x 22471697

[36] Manik SMN, Quamruzzaman M, Livermore M, Zhao C, Johnson P, Hunt I, et al. Impacts of barley root cortical aerenchyma on growth, physiology, yield components, and grain quality under field waterlogging conditions. Field Crops Research. 2022;279:108461. doi: 10.1016/j.fcr.2022.108461

[37] Pan R, Buitrago S, Feng X, Hu A, Zhou M, Zhang W. Ethylene regulates aerenchyma formation in cotton under hypoxia stress by inducing the accumulation of reactive oxygen species. Environmental and Experimental Botany. 2022;197:104826. doi: 10.1016/j.envexpbot.2022.104826

[38] Xu L, Zhao C, Pang J, Niu Y, Liu H, Zhang W, et al. Genome-wide association study reveals quantitative trait loci for waterlogging-triggered adventitious roots and aerenchyma formation in common wheat. Front Plant Sci. 2022;13:1066752. doi: 10.3389/fpls.2022.1066752 36507408 PMC9727299

[39] Grimmer MK, Boyd LA, Clarke SM, Paveley ND. Pyramiding of partial disease resistance genes has a predictable, but diminishing, benefit to efficacy. Plant Pathology. 2014;64(3):748–53. doi: 10.1111/ppa.12288

[40] Evenson RE, Gollin D. Assessing the impact of the Green Revolution, 1960 to 2000. Science. 2003;300(5620):758–62. 12730592 10.1126/science.1078710

[41] Pardey PG, Chan-Kang C, Dehmer SP, Beddow JM. Agricultural R&D is on the move. Nature. 2016;537(7620):301–3. 27629624 10.1038/537301a

[42] Alston JM, Andersen MA, James JS, Pardey PG. Persistence pays: US agricultural productivity growth and the benefits from public R & D spending. 2010.

[43] R Core Team. R: A Language and Environment for Statistical Computing. 2024.

[44] Alahmad S, Smith D, Katsikis C, Aldiss Z, Brunner SM, Meer SV, et al. Phenotyping the hidden half: combining UAV phenotyping and machine learning to predict barley root traits in the field. J Exp Bot. 2025;76(17):5161–78. doi: 10.1093/jxb/eraf268 40580084 PMC12587423

[45] Bentley A. Avert global wheat crisis caused by invasion of Ukraine. 2022. 10.1038/d41586-022-00789-x 35318475

[46] Rădulescu M, Rădulescu CZ, Zbăganu G. A portfolio theory approach to crop planning under environmental constraints. Ann Oper Res. 2011;219(1):243–64. doi: 10.1007/s10479-011-0902-7

[47] Azam-Ali SN. Ninth Revolution, The: Transforming Food Systems for Good. World Scientific; 2021.

[48] Adu MO, Asare PA, Asare-Bediako E, Amenorpe G, Ackah FK, Afutu E, et al. Characterising shoot and root system trait variability and contribution to genotypic variability in juvenile cassava (Manihot esculenta Crantz) plants. Heliyon. 2018;4(6):e00665. doi: 10.1016/j.heliyon.2018.e00665 PMC6039752 30003159

[49] Adu MO, Asare PA, Yawson DO, Nyarko MA, Abdul Razak A, Kusi AK, et al. The search for yield predictors for mature field-grown plants from juvenile pot-grown cassava (Manihot esculenta Crantz). PLoS One. 2020;15(5):e0232595. doi: 10.1371/journal.pone.0232595 32374747 PMC7202627

[50] Adu MO. Causal shoot and root system traits to variability and plasticity in juvenile cassava (Manihot esculenta Crantz) plants in response to reduced soil moisture. Physiol Mol Biol Plants. 2020;26(9):1799–814. doi: 10.1007/s12298-020-00865-4 32943817 PMC7468047

[51] Acuña TLB, Wade LJ. Use of genotype x environment interactions to understand rooting depth and the ability of wheat to penetrate hard soils. Ann Bot. 2013;112(2):359–68. doi: 10.1093/aob/mcs251 23204508 PMC3698378

[52] Evenson RE, Gollin D. Assessing the impact of the green revolution, 1960 to 2000. Science. 2003;300(5620):758–62. doi: 10.1126/science.1078710 12730592

[53] Pingali PL. Green revolution: impacts, limits, and the path ahead. Proc Natl Acad Sci U S A. 2012;109(31):12302–8. doi: 10.1073/pnas.0912953109 22826253 PMC3411969

[54] Lynch JP. Steep, cheap and deep: an ideotype to optimize water and N acquisition by maize root systems. Ann Bot. 2013;112(2):347–57. doi: 10.1093/aob/mcs293 23328767 PMC3698384

[55] Bucksch A, Burridge J, York LM, Das A, Nord E, Weitz JS, et al. Image-based high-throughput field phenotyping of crop roots. Plant Physiol. 2014;166(2):470–86. doi: 10.1104/pp.114.243519 25187526 PMC4213080

[56] Saski CA, Bhattacharjee R, Scheffler BE, Asiedu R. Genomic resources for water yam (Dioscorea alata L.): analyses of EST-sequences, de novo sequencing and GBS libraries. PLoS One. 2015;10(7):e0134031. doi: 10.1371/journal.pone.0134031 26222616 PMC4519108

[57] Tamiru M, Natsume S, Takagi H, White B, Yaegashi H, Shimizu M, et al. Genome sequencing of the staple food crop white Guinea Yam enables the development of a molecular marker for sex determination. BMC Biol. 2017;15(1):86. doi: 10.1186/s12915-017-0419-x 28927400 PMC5604175

[58] Siadjeu C, Pucker B, Viehöver P, Albach DC, Weisshaar B. High contiguity de novo genome sequence assembly of Trifoliate Yam (Dioscorea dumetorum) using long read sequencing. Genes (Basel). 2020;11(3):274. doi: 10.3390/genes11030274 32143301 PMC7140821

[59] Bellinger MR, Paudel R, Starnes S, Kambic L, Kantar MB, Wolfgruber T, et al. Taro genome assembly and linkage map reveal QTLs for resistance to Taro leaf blight. G3 (Bethesda). 2020;10(8):2763–75. doi: 10.1534/g3.120.401367 32546503 PMC7407455

[60] Yin J, Jiang L, Wang L, Han X, Guo W, Li C, et al. A high-quality genome of taro (Colocasia esculenta (L.) Schott), one of the world’s oldest crops. Mol Ecol Resour. 2021;21(1):68–77. doi: 10.1111/1755-0998.13239 32790213

[61] Prochnik S, Marri PR, Desany B, Rabinowicz PD, Kodira C, Mohiuddin M, et al. The cassava genome: current progress, future directions. Trop Plant Biol. 2012;5(1):88–94. doi: 10.1007/s12042-011-9088-z 22523606 PMC3322327

[62] Bredeson JV, Lyons JB, Prochnik SE, Wu GA, Ha CM, Edsinger-Gonzales E, et al. Sequencing wild and cultivated cassava and related species reveals extensive interspecific hybridization and genetic diversity. Nat Biotechnol. 2016;34(5):562–70. doi: 10.1038/nbt.3535 27088722

[63] Rabbi I, Hamblin M, Gedil M, Kulakow P, Ferguson M, Ikpan AS, et al. Genetic mapping using genotyping‐by‐sequencing in the clonally propagated cassava. Crop Science. 2014;54(4):1384–96. doi: 10.2135/cropsci2013.07.0482

[64] Kayondo SI, Pino Del Carpio D, Lozano R. Genome-wide association mapping and genomic prediction unravels CBSD resistance in a Manihot esculenta breeding population. bioRxiv. 2017. doi: 10.1101/158543 PMC5784162 29367617

[65] Sesay JV, Lebbie A, Wadsworth R, Nuwamanya E, Bado S, Norman PE. Genetic structure and diversity study of cassava (*Manihot esculenta*) germplasm for African Cassava Mosaic Disease and fresh storage root yield. OJGen. 2023;13(01):23–47. doi: 10.4236/ojgen.2023.131002

[66] Lebot V, Ivančič A. Taro (Colocasia esculenta (L.) Schott), breeding history, objectives, methods and strategies: a review of fifty years of sporadic efforts. Euphytica. 2022;218(11). doi: 10.1007/s10681-022-03118-5

[67] Okereke NR. Taro leaf blight: threat to taro (Colocasiae esculenta L. Schott) production. Nigeria Agricultural Journal. 2020;51(2):281–6.

[68] Hahn SK, Terry ER, Leuschner K, Akobundu IO, Okali C, Lal R. Cassava improvement in Africa. Field Crops Research. 1979;2:193–226.

[69] Hahn SK, Isoba JCG, Ikotun T. Resistance breeding in root and tuber crops at the International Institute of Tropical Agriculture (IITA), Ibadan, Nigeria. Crop Protection. 1989;8(3):147–68. doi: 10.1016/0261-2194(89)90022-7

[70] Igbozurike MU. Ecological balance in tropical agriculture. Geographical Review. 1971;61(4):519. doi: 10.2307/213390

[71] Baumüller H. Agricultural service delivery through mobile phones: local innovation and technological opportunities in Kenya. Technological and Institutional Innovations for Marginalized Smallholders in Agricultural Development. Springer;2016. p. 143–62. doi: 10.1007/978-3-319-25718-1_9

[72] Karanja L, Gakuo S, Kansiime M. Impacts and challenges of ICT based scale-up campaigns: Lessons learnt from the use of SMS to support maize farmers in the UPTAKE project, Tanzania. 2020.

[73] Kiberiti BS, Sanga CA, Mussa M, Tumbo SD, Mlozi MRS, Haug R. Farmers’ access and use of mobile phones for improving the coverage of agricultural extension service. Environmental and Agricultural Informatics. IGI Global; 2020. p. 661–86. doi: 10.4018/978-1-5225-9621-9.ch030

[74] Ortiz-Crespo B, Steinke J, Quirós CF, van de Gevel J, Daudi H, Gaspar Mgimiloko M, et al. User-centred design of a digital advisory service: enhancing public agricultural extension for sustainable intensification in Tanzania. International Journal of Agricultural Sustainability. 2020;19(5–6):566–82. doi: 10.1080/14735903.2020.1720474

[75] Lipton M. The place of agricultural research in the development of sub-Saharan Africa. World Development. 1988;16(10):1231–57. doi: 10.1016/0305-750x(88)90088-5

[76] Tadele Z, Assefa K. Increasing food production in africa by boosting the productivity of understudied crops. Agronomy. 2012;2(4):240–83. doi: 10.3390/agronomy2040240

[77] Tadele Z. Role of crop research and development in food security of Africa. International Journal of Plant Biology and Research. 2014;2(3):1019.

[78] Nassar NMA, Ortiz R. Cassava improvement: challenges and impacts. J Agric Sci. 2006;145(2):163–71. doi: 10.1017/s0021859606006575

