# Supplementary material for: Research neglect of root system architecture in root and tuber crops important for Sub-Saharan African Food Security
Source: PLoS One. 2026 Sep 9;21(9):e0357467. doi: 10.1371/journal.pone.0357467 (PMC13557360; doi:10.1371/journal.pone.0357467)
Supplement: Supplementary file 1 [file pone.0357467.s002.docx]

## **S1. RCDI classification robustness**

The “high-dependency” classification (RCDI ≥ 40%) identifies eight countries. Re-running the classification at neighbouring thresholds shows the result is stable and the country ranking does not change: lowering the cut to 30% adds four countries and raising it to 50% retains the same five at the top. RCDI is best read as a continuous measure; the 40% cut is a descriptive convenience, not a statistical boundary.

**S1 Table. Number and membership of high-dependency countries across RCDI thresholds (country-level means, 2018–2022).** Robustness of the high-dependency classification to the choice of RCDI cut-off.

| **RCDI threshold** | **Countries** | **Membership** |
| --- | --- | --- |
| ≥ 20% | **25** | Broadest cut - over half of SSA countries qualify |
| ≥ 30% | **12** | Adds Benin (37.7), Mozambique (36.8), Nigeria (34.7), Zambia (34.4) |
| ≥ 40% (used in paper) | **8** | DRC (77.4), Congo (61.4), Burundi (59.3), Ghana (56.4), CAR (51.7), Angola (49.1), Côte d’Ivoire (44.9), Rwanda (44.5) |
| ≥ 50% | **5** | DRC, Congo, Burundi, Ghana, CAR |
