# Supplementary material for: Research neglect of root system architecture in root and tuber crops important for Sub-Saharan African Food Security
Source: PLoS One. 2026 Sep 9;21(9):e0357467. doi: 10.1371/journal.pone.0357467 (PMC13557360; doi:10.1371/journal.pone.0357467)
Supplement: Supplementary file 2 [file pone.0357467.s003.docx]

## S2. One-way sensitivity of investment returns

Because the return-on-investment figures are model projections rather than measured outcomes, we examined how they respond to less-optimistic values of the three assumptions the analysis is most sensitive to: farmer adoption, farmgate price, and realised yield gain. Returns scale approximately linearly with these benefit drivers, so the table reports the headline ROI under each adjustment. Even when benefits are halved overall, the priority-focus and moderate scenarios remain near 4–5:1, consistent with the statement retained in the Discussion. We present these as scenario illustrations, not forecasts.

**S2 Table. Illustrative one-way sensitivity of ROI to benefit-side assumptions.** "Combined" corresponds to roughly a 50% reduction in realised benefits.

| **Scenario** | **Base ROI** | **Adoption −50%** | **Price −25%** | **Combined (~50% benefits)** |
| --- | --- | --- | --- | --- |
| Basic RSA | 5:1 | 2.5:1 | 3.8:1 | 2.5:1 |
| Moderate RSA | 8:1 | 4.0:1 | 6.0:1 | 4.0:1 |
| Advanced RSA | 8:1 | 4.0:1 | 6.0:1 | 4.0:1 |
| Priority focus | 10:1 | 5.0:1 | 7.5:1 | 5.0:1 |
