# Supplementary material for: Research neglect of root system architecture in root and tuber crops important for Sub-Saharan African Food Security
Source: PLoS One. 2026 Sep 9;21(9):e0357467. doi: 10.1371/journal.pone.0357467 (PMC13557360; doi:10.1371/journal.pone.0357467)
Supplement: Supplementary file 3 [file pone.0357467.s004.docx]

## S3. Regional aggregation - unweighted and population-weighted means

Regional figures in the main text use unweighted country means, treating each country as an equal policy unit so that small, highly dependent countries are not masked by a few populous neighbours. We also computed population-weighted estimates (weights: World Bank total population, 2020 midyear estimates). Weighting raises the sub-Saharan African figure rather than lowering it, because the most populous countries (Nigeria, DR Congo) are also among the most root-and-tuber-dependent. The unweighted figures reported in the paper are therefore the more conservative of the two.

**S3 Table. Mean root-and-tuber caloric supply (kcal capita⁻¹ day⁻¹) by region, unweighted versus population-weighted.** Population weights are World Bank World Development Indicators total population, 2020 midyear estimates.

| **Region** | **Unweighted** | **Population-weighted** |
| --- | --- | --- |
| Central Africa | 603.5 | 994.1 |
| East Africa | 307.7 | 273.0 |
| Southern Africa | 144.2 | 79.6 |
| West Africa | 381.8 | 571.4 |
| **Sub-Saharan Africa (all 46)** | **367.1** | **491.2** |

Population figures are World Bank World Development Indicators (2020); the weighting is dominated by the largest countries, so the comparison is insensitive to small differences in the smaller countries’ populations.
